# A Systems-Based Analysis of Mono- and Combination Therapy for Carbapenem-Resistant Klebsiella pneumoniae Bloodstream Infections

**DOI:** 10.1128/aac.00591-22

**Published:** 2022-09-20

**Authors:** Courtney L. Luterbach, Hongqiang Qiu, Patrick O. Hanafin, Rajnikant Sharma, Joseph Piscitelli, Feng-Chang Lin, Jenni Ilomaki, Eric Cober, Robert A. Salata, Robert C. Kalayjian, Richard R. Watkins, Yohei Doi, Keith S. Kaye, Roger L. Nation, Robert A. Bonomo, Cornelia B. Landersdorfer, David van Duin, Gauri G. Rao

**Affiliations:** a Division of Pharmaceutics and Experimental Therapeutics, Eshelman School of Pharmacy, University of North Carolina, Chapel Hill, North Carolina, USA; b Department of Biostatistics, Gillings School of Global Public Health, University of North Carolina, Chapel Hill, North Carolina, USA; c Centre for Medicine Use and Safety, Monash Institute of Pharmaceutical Sciences, Monash University, Melbourne, Victoria, Australia; d Department of Infectious Diseases, Cleveland Clinic, Cleveland, Ohio, USA; e Division of Infectious Diseases and HIV Medicine, Department of Medicine, Case Western Reserve University School of Medicine, Cleveland, Ohio, USA; f Department of Medicine, MetroHealth Medical Center, Cleveland, Ohio, USA; g Department of Medicine, Northeast Ohio Medical University, Rootstown, Ohio, USA; h Division of Infectious Diseases, University of Pittsburgh School of Medicine, Pittsburgh, Pennsylvania, USA; i Departments of Microbiology and Infectious Diseases, Fujita Health University School of Medicine, Aichi, Japan; j Division of Infectious Diseases, University of Michigan, Ann Arbor, Michigan, USA; k Drug Delivery, Disposition and Dynamics, Monash Institute of Pharmaceutical Sciences, Monash University, Melbourne, Victoria, Australia; l Louis Stokes Cleveland Department of Veterans Affairs Medical Center, Cleveland, Ohio, USA; m Department of Medicine, Case Western Reserve University School of Medicine, Cleveland, Ohio, USA; n Departments of Pharmacology, Molecular Biology and Microbiology, Biochemistry, and Proteomics and Bioinformatics, Case Western Reserve University School of Medicine, Cleveland, Ohio, USA; o CWRU-Cleveland VAMC Center for Antimicrobial Resistance and Epidemiology (Case VA CARES), Cleveland, Ohio, USA; p Division of Infectious Diseases, University of North Carolina, Chapel Hill, North Carolina, USA; q Department of Pharmacy, Fujian Medical University Union Hospital, Fuzhou, Fujian, People’s Republic of China; r Institute for Global Health and Infectious Diseases, University of North Carolina, Chapel Hill, North Carolina, USA

**Keywords:** ceftazidime-avibactam, colistin, Enterobacterales, pharmacodynamics, machine learning

## Abstract

Antimicrobial resistance is a global threat. As “proof-of-concept,” we employed a system-based approach to identify patient, bacterial, and drug variables contributing to mortality in patients with carbapenem-resistant Klebsiella pneumoniae (CR*Kp*) bloodstream infections exposed to colistin (COL) and ceftazidime-avibactam (CAZ/AVI) as mono- or combination therapies. Patients (*n* = 49) and CR*Kp* isolates (*n* = 22) were part of the Consortium on Resistance Against Carbapenems in Klebsiella and other Enterobacteriaceae (CRACKLE-1), a multicenter, observational, prospective study of patients with carbapenem-resistant Enterobacterales (CRE) conducted between 2011 and 2016. Pharmacodynamic activity of mono- and combination drug concentrations was evaluated over 24 h using *in vitro* static time-kill assays. Bacterial growth and killing dynamics were estimated with a mechanism-based model. Random Forest was used to rank variables important for predicting 30-day mortality. Isolates exposed to COL+CAZ/AVI had enhanced early bacterial killing compared to CAZ/AVI alone and fewer incidences of regrowth compared to COL and CAZ/AVI. The mean coefficient of determination (R^2^) for the observed versus predicted bacterial counts was 0.86 (range: 0.75 − 0.95). Bacterial subpopulation susceptibilities and drug mechanistic synergy were essential to describe bacterial killing and growth dynamics. The combination of clinical (hypotension), bacterial (IncR plasmid, *aadA2*, and *sul3*) and drug (KC_50_) variables were most predictive of 30-day mortality. This proof-of-concept study combined clinical, bacterial, and drug variables in a unified model to evaluate clinical outcomes.

## INTRODUCTION

Bacterial resistance to antibiotics is outpacing antibiotic development. Resistant microorganisms already cause ~2.8 million infections annually in the United States (1). Carbapenem-resistant Enterobacterales (CRE) belong to the ESKAPE group of nosocomial pathogens that are notorious for evading antibiotics (2). Drug selection is further complicated by limited knowledge of the optimal therapeutic dose and duration to inhibit bacterial growth at sites of infection (3). Subtherapeutic regimens select for more resistant bacteria, requiring the use of higher drug concentrations (4). Illness severity among other variables also impacts drug pharmacokinetic (PK) profiles and efficacy (5). Conventional drug selection approaches overlook the complex patient-bacteria-drug environment. Thus, system-based approaches incorporating drug PK/pharmacodynamic (PD) metrics with bacterial and patient characteristics may better model infections and help optimize drug regimens.

Historically, aminoglycosides, polymyxins (colistin [COL] and polymyxin B), tigecycline, and fosfomycin were commonly administered to treat CRE infections; however, bacterial resistance rates limit their use (2). Adverse effect profiles, such as nephrotoxicity and neurotoxicity, can also hamper dose escalation strategies for achieving therapeutic concentrations (6). Ceftazidime-avibactam (CAZ/AVI) is an extended-spectrum cephalosporin and a novel non-β-lactam β-lactamase inhibitor (7). AVI inhibits Ambler class A, C, and some D β-lactamases and can thereby restore the activity of CAZ (8). Several clinical studies found that patients receiving regimens that included CAZ/AVI, compared to other regimens (e.g., COL, carbapenem [CB], CB + aminoglycoside, CB+COL) for treatment of CRE infections, had improved or comparable clinical success (9–11). Combination therapy (e.g., CAZ/AVI-COL, CAZ/AVI-aztreonam) has shown promise for treating MDR infections (12–14), although evidence from randomized controlled trials (RCT) remains limited. Testing newer combination regimens against clinical isolates is restricted by cost, time, and ethical concerns. Also, stringent inclusion and exclusion criteria for selecting participants can narrow the generalizability of clinical findings.

Machine learning (ML) algorithms ‘learn’ patterns from large heterogeneous data sets and have assisted with antibiotic selection and predicting factors involved with disease progression (e.g., heart disease and diabetes) (15). ML can also provide initial covariate selection for pharmacometric models (16–18). The union of these approaches improves data-driven decision making and enables the timely evaluation of a greater number of covariates. With increasing accessibility to electronic health care and genomic data, ML has the potential to uncover novel predictive signatures and cross-disciplinary interactions between variables (19). As “proof-of-concept,” here we applied a systems-based ML approach, which combines clinical, bacterial, and mechanism-based modeling variables in a unified model to predict mortality in patients with carbapenem-resistant K. pneumoniae (CR*Kp*) bloodstream infections (BSI) treated with either COL or CAZ/AVI alone, or as combination therapy.

## RESULTS

### Patient variables.

**FIG 1:**
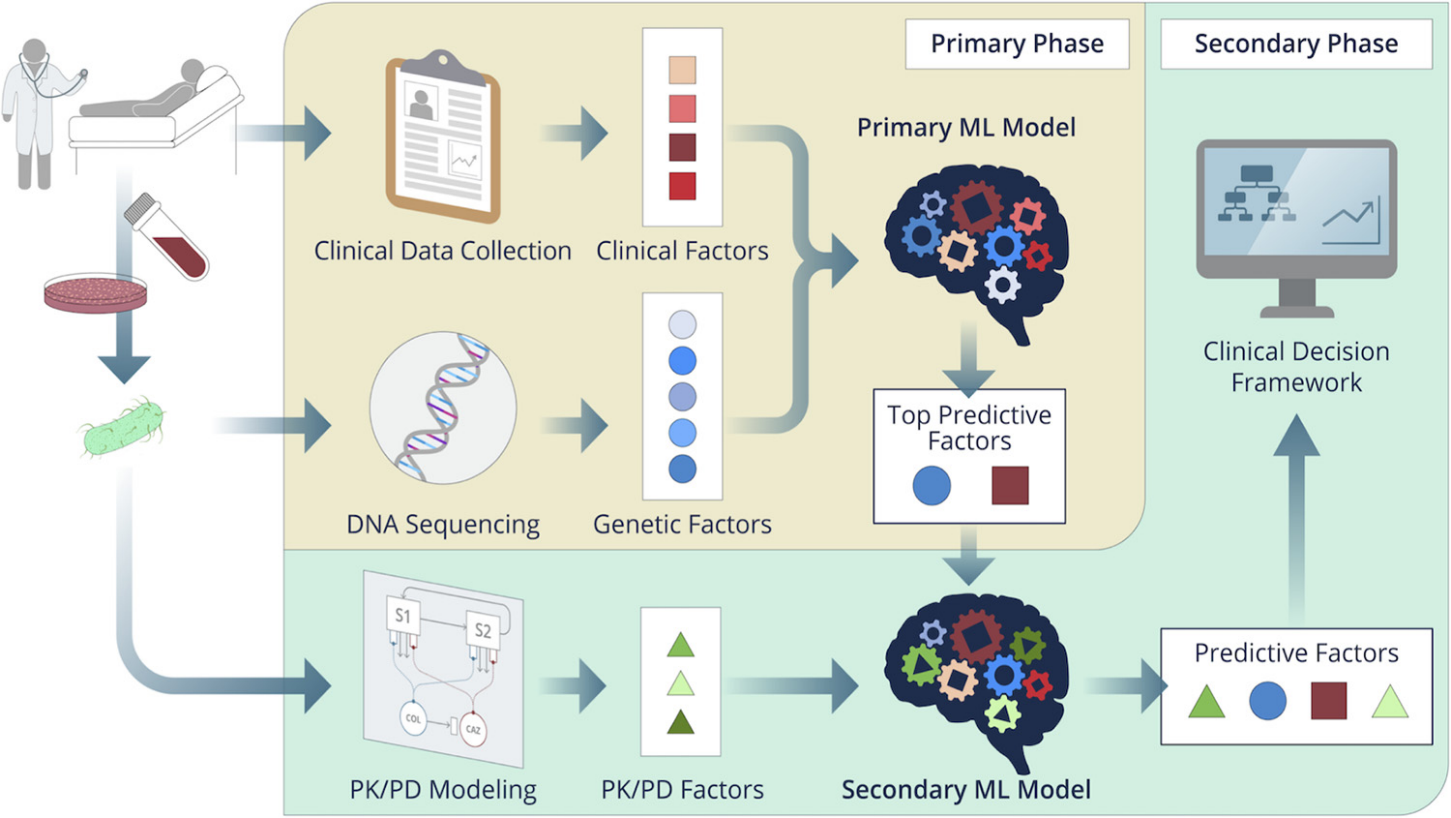
Schematic of the systems-based approach that combines variables derived from clinical data, bacterial genetic analysis, and mechanism-based modeling into a machine learning model to predict variables impacting 30-day mortality in patients with CR*Kp* BSI treated either individually or in combination with colistin and ceftazidime-avibactam.

A schematic of our systems-based approach is shown in Fig. 1. Baseline patient characteristics are summarized in Table 1. Out of the total 966 CRACKLE-1 patients, 174 had a CR*Kp* BSI and 49 met the inclusion criteria for this study. Most patients in the primary cohort were treated with COL monotherapy (59%) and either acutely or chronically ill with a median Pitt bacteremia score of 4 (interquartile range (IQR): 2, 4) and Charlson comorbidity index of 3 (IQR: 2, 5). About half the patients (*n* = 15/29, 52%) treated with COL monotherapy died within 30 days of index culture compared to only 9% (*n* = 1/11) of patients treated with CAZ/AVI. None of the patients receiving combination therapy died (*n* = 0/9). Of the patients receiving combination therapy, five (56%) received COL first, one (11%) received CAZ/AVI first, and three (33%) received COL and CAZ/AVI on the same day. Exposure to multiple antibiotics was common among patients (Table S2). Baseline characteristics of the secondary patient subcohort are summarized in Table S3.

**TABLE 1:** Baseline clinical characteristics of primary cohort

Characteristic, *n* or median (% or IQR)	30-day mortality		All patients (*n* = 49)
	No (*n* = 33)	Yes (*n* = 16)	
Sex; female	16 (49)	9 (56)	25 (51)
Race			
Caucasian	13 (39)	9 (56)	22 (45)
African American	16 (49)	7 (44)	23 (47)
Other	4 (12)	0 (0)	4 (8)
Ethnicity			
Not Hispanic or Latino	31 (94)	15 (94)	46 (94)
Unknown	2 (6)	1 (6)	3 (6)
Age (yrs)	63 (50, 73)	68 (58, 76)	66 (51, 75)
Highest creatinine level (mg/dL)^*a*^	2.0 (1.0, 3.0)	2.0 (1.0, 3.3)	2.0 (1.0, 3.0)
Highest neutrophil count (1,000 cells/μL)^*a*^	10 (3.5, 17)	12 (11, 18)	12 (8.0, 17)
Lowest hemoglobin level (g/dL)^*a*^	9.0 (8.0, 10)	9.0 (8.0, 10)	9.0 (8.0, 10)
Highest peripheral white blood cell count (1,000 cells/μL)^*c*^	12 (6.0, 19)	18 (12, 28)	13 (9.0, 21)
Highest temp (°C)^*a*^	37.9 (37.0, 38.5)	37.5 (37.0, 38.3)	37.7 (37.0, 38.5)
Immunocompromised	8 (25)	3 (21)	11 (24)
Congestive heart failure	8 (24)	7 (47)	15 (31)
Peripheral vascular disease	3 (9)	3 (20)	6 (13)
Cerebrovascular disease	6 (18)	3 (20)	9 (19)
Diabetes mellitus	16 (49)	6 (40)	22 (46)
Malignancy within last 5 yrs	8 (24)	3 (20)	11 (23)
Chronic kidney disease	10 (30)	7 (47)	17 (36)
Cirrhosis	4 (12)	3 (20)	7 (15)
Renal failure	17 (52)	12 (75)	29 (59)
Pitt bacteremia score	2 (2, 4)	4 (4, 5)	4 (2, 4)
Charlson comorbidity index	3 (2, 5)	5 (3, 6)	3 (2, 5)
History of coronary artery disease/myocardial infarction	9 (27)	3 (20)	12 (25)
Hypotension	19 (58)	14 (88)	33 (67)
Surgery	8 (24)	5 (31)	13 (27)
Central venous line	21 (66)	15 (94)	36 (75)
Mechanical ventilation	13 (39)	13 (81)	26 (53)
Treatment			
COL	14 (42)	15 (94)	29 (59)
CAZ/AVI	10 (30)	1 (6)	11 (22)
COL+CAZ/AVI	9 (27)	0 (0)	9 (18)
Time to CAZ/AVI treatment (days)^*b*^	3 (2, 4)	0 (0, 0)	3 (2, 4)
Time to COL treatment (days)^*b*^	3 (1, 3)	2 (0, 2)	2 (1, 3)
Time from admission to index culture (days)	0 (0, 4)	1 (0, 18)	0 (0,10)

a Recorded on date of index CR*Kp* blood culture.

b Time to treatment indicates the number of days between index CR*Kp* blood culture and receiving drug treatment.

c IQR, interquartile range; COL, colistin; CAZ/AVI, ceftazidime-avibactam.

### Bacterial variables.

Most CR*Kp* were sequence type (ST) 258 (73%) followed by ST11 (4%) and ST307 (4%) (Table 2, Fig. S2). Predominant capsular types by *wzi* sequencing were 154 (43%) and 29 (35%), and the most frequent carbapenemases were KPC-2 (47%), KPC-3 (50%), and OXA-232 (6%). Baseline characteristics for isolates from the secondary patient subcohort were similar in distribution to those from the primary cohort (Table S4). Most isolates in the secondary subcohort were susceptible to both COL (median MIC: 0.25 mg/L, range: 0.25 to 32 mg/L) and CAZ/AVI (median MIC: 1 mg/L, range: 0.25 to 4 mg/L).

**TABLE 2:** Baseline bacterial characteristics of the primary cohort

Characteristic, *n* (%)^*e*^	30-day mortality		All patients (*n* = 49)
	No (*n* = 33)	Yes (*n* = 16)	
Sequence Type			
258	24 (73)	12 (75)	36 (73)
11	0 0	2 (13)	2 (4)
307	2 (6)	0	2 (4)
Other^*a*^	7 (21)	2 (13)	9 (18)
*wzi* capsule type			
154	13 (39)	8 (50)	21 (43)
29	12 (36)	5 (31)	17 (35)
173	2 (6)	0	2 (4)
27	0	2 (13)	2 (4)
Other	6 (18)	1 (6)	7 (14)
Carbapenemase^*b*^			
*bla*_KPC-2_	15 (45)	8 (50)	23 (47)
*bla*_KPC-3_	16 (50)	8 (50)	24 (50)
*bla*_OXA-232_	2 (6)	1 (6)	3 (6)
ESBL			
*bla*_SHV-12_	15 (45)	10 (63)	25 (51)
*bla*_CTX-M_^*c*^	8 (24)	2 (13)	10 (20)
Antimicrobial nonsusceptibility^*d*^			
COL	2/31 (6)	1/15 (7)	3/46 (7)
CAZ/AVI	0/20	0/12	0/32

a Includes ST16(1), ST231(1), ST2891(1), ST3631(1), ST37(1), ST418(1), ST45(1), ST76(1), ST985(1).

b Isolates could carry multiple genes encoding β-lactamases.

c Includes *bla*_CTX-M-14_ (1) and *bla*_CTX-M-15_ (9).

d Includes only those isolates tested for susceptibilities.

e ESBL, extended-spectrum β-lactamase; COL, colistin; CAZ/AVI, ceftazidime-avibactam.

### Mechanism-based modeling.

**FIG 2:**
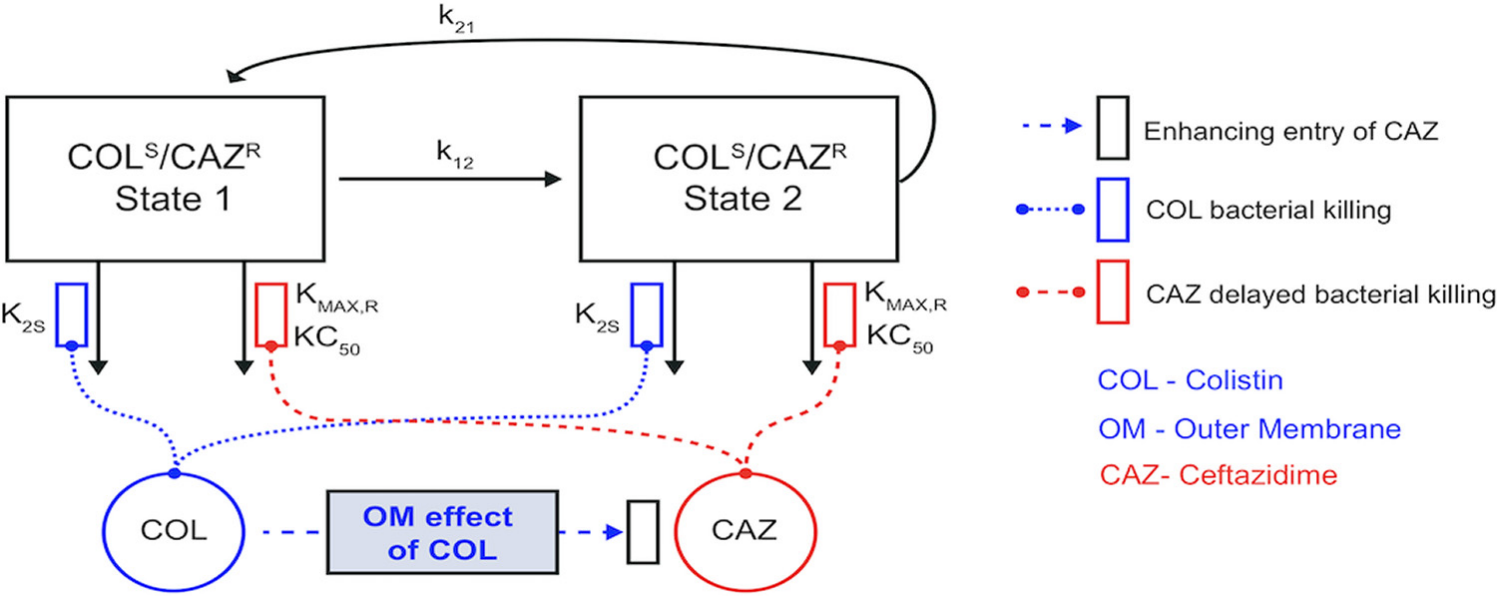
Schematic of the mechanism-based bacterial life cycle model depicting the shift in ceftazidime (CAZ) (red) KC_50_ through colistin (COL) (blue) disruption of the outer bacterial membrane for the COL-susceptible/CAZ-resistant subpopulation. Transit compartments, not depicted in this figure, were also included in the model to represent the inhibition of cell wall synthesis during bacterial replication caused by CAZ inhibition of penicillin-binding proteins.

To characterize the PD of each drug against CR*Kp* from the secondary patient subcohort, a range of therapeutic and supratherapeutic concentrations of COL and/or CAZ/AVI was tested in static time-kill (SCTK) assays (Fig. S3-S7). SCTK data were incorporated into a mechanism-based model to characterize bacterial killing and growth dynamics of CR*Kp* (*n* = 22) treated with COL and/or CAZ/AVI (Fig. 2). A unified model for all isolates with the same model parameters was necessary for subsequent incorporation into the ML models. The model simultaneously described the effects of COL and CAZ/AVI and overall yielded good curve fits for most concentrations (Fig. S3-S7). Time versus predicted plots show uniformly distributed predictions at each sample time (Fig. S9). The mean R^2^ for the observed versus predicted viable counts was 0.86 (min-max: 0.75 − 0.95), meaning the model was predictive of, at worst, ≥75% of the observed data for each isolate. The median CAZ concentration needed to achieve 50% of maximal killing (KC_50_) was 0.935 (range: 0.492 − 2.06) mg/L (Table S5).

Baseline population analysis profiles (Fig. S8) enabled demonstration of mutation frequencies of less-susceptible and resistant subpopulations. Subpopulation synergy alone (as described in the supplemental material) did not adequately describe the time course of bacterial load reduction and regrowth. Mechanistic synergy due to COL enhancing target site concentrations of CAZ was essential to describe the viable count profile time course. Model estimation of the synergy term, I_MAX_,_OM_ (median [range]: 0.603 [−0.264, 0.725]) demonstrates that COL+CAZ/AVI combination results in increased sensitivity to CAZ/AVI as we see a reduction in KC_50,CAZ_ with the addition of COL, and thus the combination has a synergistic antibiotic effect (Table S5 and S6). Most isolates (16/22, 73%) had a maximum fractional decrease of KC_50,CAZ_ (i.e., I_MAX,OM_ >0) denoting a synergistic killing effect.

### Machine learning model.

**FIG 3:**
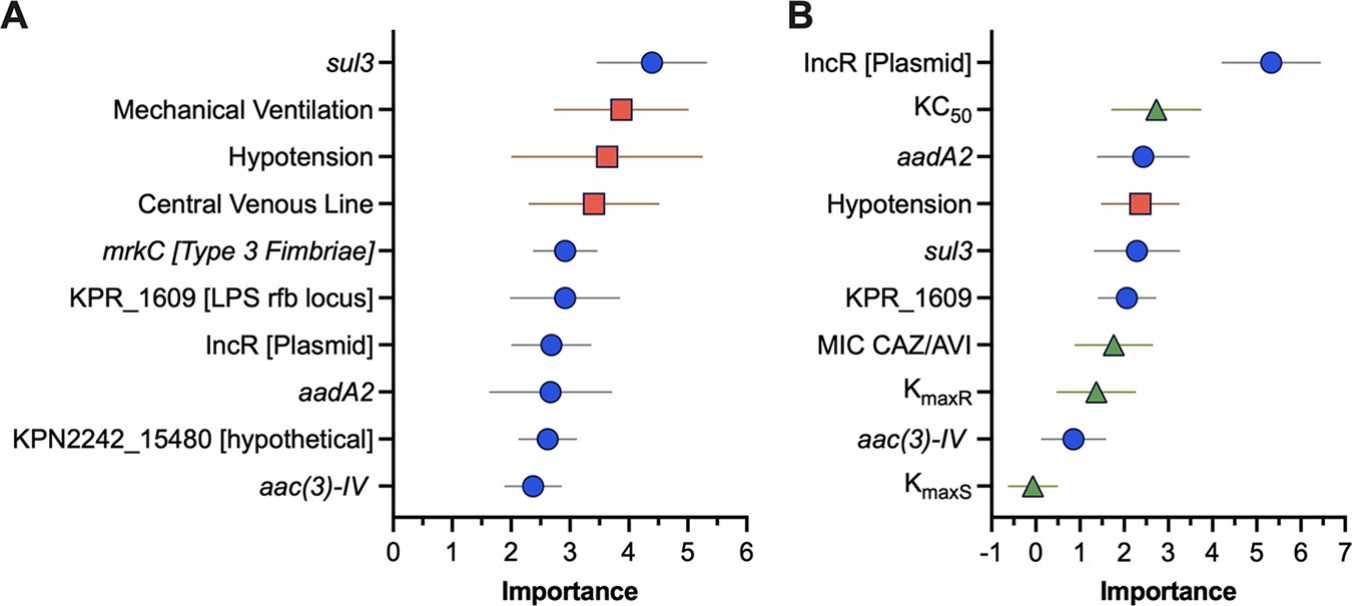
Top 10 variables identified by Random Forest in the (A) primary cohort (*n* = 49) and (B) secondary subcohort (*n* = 22) that are predictive of 30-day mortality from index CR*Kp* blood culture. Variables are ranked by the average relative importance with error shown as the 95% CI. Dot color indicates the data source for each variable (clinical [red squares], bacterial genetic [blue circles], drug [green triangles]). The primary model includes only clinical and bacterial genetic variables, while the secondary model includes clinical, bacterial genetic, and drug variables.

Random Forest (RF) was used to combine clinical, bacterial, and *in vitro* PD variables into a single model to rank the variables predicting 30-day mortality. Overall, model accuracy for the validation data set for the primary RF was 69.4%, and the top variables were carriage of *sul3*, mechanical ventilation, and hypotension (Fig. 3A). Model accuracy for the validation data set for the secondary RF was 73.0%. The top variables predicting mortality were IncR, KC_50_, and *aadA2* with a variable importance of 5.3, 2.7, 2.4, respectively (Fig. 3B). CAZ/AVI MIC and COL MIC were ranked 7th and 14th, respectively. The top bacterial variables were more common in patients who died within 30 days of the index culture (Table S7).

## DISCUSSION

Early detection of treatment failure would provide opportunities to optimize drug regimens and reduce health care costs. In general, incorporating population-based and high-dimensional data with the knowledge gained from PK/PD models is challenging. Here, we used ML to incorporate patient, bacterial, and *in vitro* drug PD variables into a unified model to identify variables predicting 30-day mortality in patients with a CR*Kp* BSI treated with COL and/or CAZ/AVI.

Overall, patients treated with COL, compared to CAZ/AVI or COL+CAZ/AVI, had higher rates of 30-day mortality. COL+CAZ/AVI combination had similar early bacterial killing as COL monotherapy while limiting regrowth. No patients receiving COL+CAZ/AVI died within 30 days, though the sample size is small. Extent of early bacterial killing is overlooked when using point-based measures to determine drug activity. However, initiation of effective antimicrobial administration within the first hour of documented hypotension was associated with increased survival in septic shock, whereas each hour of delay decreased survival by an average of 7.6% (20). Thus, we utilized a mechanism-based model to characterize bacterial killing and growth in response to a range of clinically achievable drug exposures and to rank the predicted value of MBM variables relative to baseline MIC values. Heteroresistance, where a predominantly susceptible population includes a subpopulation of less susceptible bacteria, is common among CRE (21). Bacterial killing and growth dynamics were best modeled when including antibiotic synergy and bacterial subpopulations of heterogeneous susceptibility.

Combination therapies may delay emergence of resistance, compared to monotherapies, due to in part a broader spectrum of activity and synergism (14). COL destabilizes outer membrane permeability, which may enhance entry of other antimicrobials into the intracellular space and induce mechanistic synergy (13). Synergistic activity of COL-containing combinations, including COL+CAZ/AVI, against CR*Kp* has been observed *in vitro* (13). We observed a synergistic killing effect for CAZ/AVI+COL to most CR*Kp* isolates. In contrast, Shields et al. identified synergy at 24 h in only ~19% of CR*Kp* isolates (*n* = 16) treated with COL+CAZ/AVI based on a different definition for synergy (22). Bacterial genetic diversity or differences in drug preparation and administration may explain the variability in drug responses. Further investigation to understand the sources of variability in therapies against MDR bacteria is warranted.

A strength of this study is the number of isolates included in the mechanism-based model, which are often limited to a few isolates. A unified mechanism-based model representing all isolates was employed to compare isolates against each other in the ML approach. Thus, the therapeutic response may be oversimplified (i.e., model misspecification) or too complex (i.e., overfitting) for some parameters in some isolates. Adding another subpopulation to describe intermediate resistance to COL (CRK022, CRK0091, and CRK0094) or CAZ (CRK0091) resulted in improved model fits, but COL monotherapy was not always captured at all doses effectively (CRK0098, CRK0078, and CRK0030). Two isolates had initial subpopulation sizes described with values that may be considered negligible (CRK0022 and CRK0392: Log_MF_RR_ <−10). Increased sampling in future studies may better resolve the model. However, the mechanism-based model was able to describe most isolates reasonably well. We plan to expand this work by simulating clinically relevant humanized PK exposures and the resulting PD response for patients in a ML model.

The ML model identified the IncR plasmid as the highest ranked predictor. IncR is associated with carriage of several antibiotic-resistance genes (23). Isolates with this plasmid may be more resistant to treatment and/or the replicon serves as a surrogate marker for other, untested gene(s) on the plasmid. KC_50_, the CAZ/AVI concentration necessary to achieve half-maximal killing, was also identified as a top predictor of mortality. This parameter indicates how sensitive the bacterial isolate is to CAZ/AVI, and is a more granular descriptor of the bacteria-drug relationship than MIC or the area under the bacterial growth curve. Thus, KC_50_ is both a drug- and isolate-related parameter.

Limitations of this study include that SCTK assays are limited in recapitulating *in vivo* drug PK. However, apart from a small proportion of supratherapeutic concentrations that were included to assist with improved model performance and parameter estimates, the range of concentrations used in the SCTK assay are clinically achievable and mimic steady-state concentrations previously documented in patients (24–27). Also, patients were often empirically treated with additional antimicrobials and data on dosing regimens and the achieved drug exposure were not available. Thus, the clinical outcomes may have been impacted in part by a range of drug exposures at the target site of infection. The small sample size, especially regarding patients treated with combination therapy, also limited our statistical and ML analysis. A small sample size for RF can contribute to biased performance estimates and poor pattern recognition (28). Thus, a nested CV approach was employed for the primary RF to incorporate feature selection and hyperparameter optimization in efforts to limit overoptimistic predictions during training (28). Variables identified via ML are not definitive markers for clinical management or treatment decisions but rather potential candidates for follow-up in *in silico* and *in vitro* studies.

It may not be feasible to perform bacterial genomic sequencing and determine the KC_50_ in a timely fashion during an infection. However, applying a broader application of this approach in size and isolate diversity could help identify predictors of interest for follow-up studies. For example, bacterial genes associated with treatment failure may be good candidates for developing rapid genetic screening tools to guide dosing regimens to ensure treatment efficacy. Likewise, mechanism-based models can assist the development of human population PK models with the potential to optimize individualized drug therapy (29–34). Thus, ML algorithms show promise as hypothesis-generating tools for personalized medicine and as systems-based platforms generalizable to other treatments.

## MATERIALS AND METHODS

Isolates, antibiotics, and media are described in the supplemental material.

### Patient cohort.

The Consortium on Resistance Against Carbapenems in Klebsiella and other *Enterobacteriaceae* (CRACKLE-1) was a multicenter, observational, prospective study of patients with CRE admitted to US hospitals from December 2011 to June 2016 (35). Descriptions of inclusion criteria and clinical measurements are in the supplemental material. We analyzed a primary cohort of 49 patients with a CR*Kp* BSI treated ≤10 days from index blood cultures with either mono- or combination CAZ/AVI and COL. Combination therapy was defined as administration of CAZ/AVI and COL either on the same day or at any time sequentially within 48 h. A secondary subcohort of 22 patients was selected from the primary cohort based on isolate availability for *in vitro* evaluation of PD activity of COL and CAZ/AVI at the time of the study. The secondary subcohort size was constrained to 22 isolates as this was a reasonable number of isolates (i.e., 506 treatment arms [i.e., exposures to different concentrations] in total across the 22 isolates) for static time-kill studies (SCTK) and to describe with a common mechanism-based model structure. The study was approved by institutional review boards at all sites.

### Static time-kill studies (SCTK).

The PD of COL and CAZ/AVI as mono- and combination therapies was evaluated against CR*Kp* isolates from the secondary subcohort over 24 h, as previously described ([36] and supplemental material). COL (0.5 to 16 mg/L) and CAZ/AVI concentrations (16/4 to 128/32 mg/L) were evaluated as monotherapies. A 4 × 3 matrix of COL (0.5, 1, 2, and 4 mg/L) in combination with CAZ/AVI (16/4, 32/8, and 64/16 mg/L) was also evaluated. Concentration ranges included clinically achievable and supratherapeutic unbound plasma concentrations to evaluate the potential benefit of intensive dosing (7, 24–27). Samples were obtained at 0, 1, 2, 4, 6, 8, and 24 h for bacterial quantification with the limit of quantification at 20 CFU/mL. Early bacterial killing was defined as the average decrease in log_10_ CFU/mL at 1 h. Bacterial regrowth was defined as an increase in CFU/mL from 8 to 24 h as this was the time interval that best captured any increase in bacterial load in isolates exposed to high drug concentrations.

### Mechanism-based model development.

A mechanism-based model was developed to quantify the time course of CR*Kp* killing and regrowth for COL and CAZ/AVI alone and in combination using SCTK data. The total inoculum consisted of four subpopulations to describe each isolate based on COL and CAZ susceptibility and being in either a growth or replicating life cycle (37, 38). Subpopulations were determined by model discrimination using diagnostic plots, biological feasibility of parameter estimates, and objective function values. Mechanistic synergy was described as the disruption of the bacterial outer membrane by COL resulting in increased penetration and action of CAZ on penicillin-binding proteins (PBPs) (39). Additional details are provided in the supplemental material.

### Random Forest (RF) algorithm.

RF with nested cross-validation (nCV) was used to rank by importance the patient, bacterial, and drug variables for predicting 30-day mortality (Fig. S1). First, a RF was performed incorporating only the patient and bacterial variables for patients within the primary cohort. The top 10 important variables were combined with mechanism-based model and MIC variables into a second RF model for patients within the secondary subcohort. RF variables are listed in Table S1 with additional details in the supplemental material.
