# Supplementary material for: A Systems-Based Analysis of Mono- and Combination Therapy for Carbapenem-Resistant Klebsiella pneumoniae Bloodstream Infections
Source: Antimicrob Agents Chemother. 2022 Sep 20;66(10):e00591-22. doi: 10.1128/aac.00591-22 (PMC9578421; doi:10.1128/aac.00591-22)
Supplement: Supplemental file 1 — Supplemental material. Download aac.00591-22-s0001.docx, DOCX file, 41.7 MB [file aac.00591-22-s0001.docx]

**Supplementary Materials**

**Supplemental Methods**

*Baseline Patient Characteristics*

All clinical data were obtained from electronic medical records as part of the CRACKLE-1 study. Inclusion criteria included a positive blood culture of CR*Kp*, intravenous treatment with either COL and/or CAZ/AVI within 10 days following an index positive culture, and a collected isolate for whole genome sequencing. Patients may have had additional positive CR*Kp* cultures collected from other body sites but only data from the index blood culture were included. One patient, included only in the primary cohort, died within 48 h from index culture and was administered COL on the index culture date. For analysis purposes, patients discharged to hospice within 30 days of index CRKp were considered deceased at time of discharge.

*Clinical Measurements*

Highest creatinine or neutrophil levels and lowest hemoglobin levels were recorded on the date of index culture. Immunocompromised patients were those patients with either HIV (with or without AIDS), solid organ transplant, hematopoietic stem cell transplant, receiving prednisone 10 mg/day or an equivalent steroid, or received TNF-α blocker or monoclonal antibody treatment within the past month. Chronic kidney disease was defined as a history of kidney disease and a serum creatinine level $\geq$2 mg/dL. Renal failure was defined as a serum creatinine level $\geq$2 mg/dL and/or the use of renal replacement therapy. Malignancy was a diagnosis of either solid tumor, leukemia or lymphoma. The Pitt bacteremia score (1) and Charlson Comorbidity Index (2) were calculated as described previously. Hypotension was defined as a drop in systolic blood pressure >30 mm Hg and diastolic blood pressure >20 mm Hg, requirement for intravenous pressor agents, or systolic blood pressure <90 mm Hg. PK data were not collected as part of the CRACKLE-I study, and therefore it was not possible to calculate exposure metrics for individual subjects.

*Bacterial isolates, antibiotics, and media*

CR*Kp* were identified based on Clinical and Laboratory Standards Institute (CLSI) guidelines. Antimicrobial susceptibility testing was previously performed using MicroScan (Siemans Diagnostics) or Vitek2 (bioMérieux) and GN4F Sensititre tray (Thermo Fisher Scientific) or Etest (bioMérieux) (3). We independently determined the minimum inhibitory concentration (MIC) for CAZ/AVI and COL by broth microdilution according to CLSI (4) and EUCAST (5) guidelines, respectively. MIC determination and *in vitro* static time-kill studies were performed using cation-adjusted (25.0 mg/L Ca^2+^ and 12.5 mg/L Mg^2+^) Mueller-Hinton broth (CAMHB; Becton, Dickinson and Company, Sparks, MD). Stock solutions of COL (Sigma-Aldrich, St Louis, MO), CAZ hydrate (Sigma-Aldrich), and AVI (Advanced ChemBlocks Inc., Burlingame, CA) were prepared in water and filter sterilized through 0.22-µm syringe filters (Corning Inc., Corning, NY) immediately prior to each experiment. Baseline mutation frequencies were determined via population-analysis profiles (PAPs). Samples (200μL) of a bacterial inoculum prepared from overnight growth were plated on cation-adjusted (25.0 mg/L Ca^2+^ and 12.5 mg/L Mg^2+^) Mueller-Hinton agar (CAMHA; Becton, Dickinson and Company, Sparks, MD), supplemented with the required antibiotic concentrations (6). Plates were incubated at 37°C for 24 h (up to 48 h for smaller colonies), bacterial colonies were counted, and the CFU/mL were determined.

Carbapenemase genes were previously detected by polymerase chain reaction and confirmed with whole genome sequencing (3). Screening for virulence and antimicrobial resistance genes was performed using ABRicate (<https://github.com/tseemann/abricate>) with the CARD (7), ResFinder (8), VFDB (9), and PlasmidFinder (10) databases. Genes with more than 90% sequence coverage and 95% sequence identity with the reference gene sequences were defined as being present in the genome. WGS data are publicly available on NCBI (PRJNA433394, PRJNA339843).

*SNP alignment, MLST typing, and wzi typing*

Trimmed paired-end sequences for each genome were mapped to ST258 reference strain NJST258_2 (11). Single nucleotide polymorphisms (SNPs) were mapped using snippy (https://github.com/tseemann/snippy) using the default parameters. DNA regions identified as prophages (PHASTER) (12), duplications (MUMmer) (13) and sites of recombination (Gubbins) (14) were masked from the core SNP alignment. MLST and *wzi* typing were conducted using MLST (<https://github.com/tseemann/mlst>) and Kleborate (15), respectively.

*Dendrogram construction*

FastTree (16) was used to build an maximum-likelihood phylogenetic tree from the core SNP alignment using a generalized time-reversible model of nucleotide evolution. Support values for nodes were estimated by performing bootstrapping analysis (n=1000). FigTree (17) and Circos (18) were used for visualization of the tree and associated genomic and clinical metadata.

*Static time-kill studies (SCTK)*

The pharmacodynamic activity of COL and CAZ/AVI as mono and combination therapy was evaluated against 22 CR*Kp* isolates from the secondary cohort over 24 h as previously described (19). COL concentrations of (0.5, 1, 2, 4, 8, and 16 mg/L) and CAZ concentrations in combination with AVI at a fixed ratio of 4 to 1 (16/4, 32/8, 64/16, and 128/32 mg/L) were evaluated as monotherapy. A 4x3 matrix of COL (0.5, 1, 2, and 4 mg/L) in combination with CAZ/AVI (16/4, 32/8, and 64/16 mg/L) with an untreated growth control were also evaluated. By characterizing each drug at both therapeutic and supratherapeutic concentrations, there is higher certainty that we are able to determine the maximal killing ability of the antibiotic, and this assists with improved model performance and parameter estimates with improved precision (20, 21). The steady state average concentration achieved with total COL ranges from 0.48 to 9.38 mg/L for doses ranging from 75 to 410 mg/day dosed every 8 to 24 hours, including in patients on renal replacement therapy (22-27). AVI dosed as a 500 mg 2-hour intravenous infusion every 8 h yields plasma concentrations ranging from 3.8 to 5.7 mg/L, and the steady-state average concentrations following infusion with 2000 mg of CAZ every 8 hours range from 31 to 42 mg/L (25, 26). Protein binding ratios for COL, CAZ, and AVI are approximately 50, 10, and 8.2% respectively (24, 25).

Bacterial colonies were cultured in CAMHB to an initial inoculum of 10^6^ colony forming units (CFU/mL). Cultures were then incubated in a water bath at 37°C with constant shaking, and antibiotic(s) were added to the culture in the logarithmic growth phase. Samples were obtained at 0, 1, 2, 4, 6, 8, and 24 h for bacterial quantification. A ProtoCOL automated colony counter (Symbiosis, Cambridge, United Kingdom) was used to quantify the bacteria following 24 h of incubation at 37°C. The limit of detection was 20 CFU/mL. Statistical differences were compared using Kruskal-Wallis test with secondary testing using Dunn's multiple comparisons test (**Figure S4**).

Early bacterial killing at 1 h was greater for isolates treated with either COL (-3.10 log_10_ CFU, p<0.001) or COL+CAZ/AVI (-2.88 log_10_ CFU, p<0.001) compared to CAZ/AVI alone (-0.72 log_10_ CFU) (**Figure S3**). Most strains treated with the highest concentration of COL (16 mg/L) (91%, n=20/22) or COL+CAZ/AVI (4mg/L+64/16mg/L) (95%, n=21/22) had >3 log_10_ reduction in bacterial burden by 4 h, compared to CAZ/AVI alone (128/32 mg/L) (55%, n=12/22) **(Figures S4-S7)**. Regrowth was observed in 95% (n=21/22) of strains treated with the highest concentration of COL (16 mg/L), compared to 32% (n=7/22) and 9% (n=2/22) for CAZ/AVI (128/32 mg/L) and COL+CAZ/AVI (4mg/L+64/16mg/L), respectively. COL+CAZ/AVI treatment resulted in lower bacterial counts at 24 h than COL (1.4 vs. 5.1 log_10_ CFU/mL, p=0.0004) and CAZ/AVI (1.4 vs 1.9 log_10_ CFU/mL, p=0.049).

*Mechanism-based model development*

A mechanism-based model (MBM) was developed to quantify the time course of bacterial killing and regrowth for both COL and CAZ/AVI alone and in combination against CR*Kp* using the SCTK data. Several modeling publications were consulted including Ly et al. (28), Bulitta et al. (29), and Rao et al. (30). To account for heterogeneity in the bacterial inoculum, a life-cycle growth model was employed in which the total inoculum was characterized by subpopulations based on their susceptibility to COL and CAZ (28, 30-33). Models with two, three, and four subpopulations were explored (34). For each subpopulation, the bacteria life cycle consists of two states: a growth state and a replicating state (35). Bacterial subpopulations describing the bacterial inoculum, bacterial killing terms (first-order and sigmoidal killing terms), and transit compartments describing CAZ effect were explored and determined by performing model discrimination using diagnostic plots, biological feasibility of the parameter estimates, and objective function values. The final model described the total bacteria load (CFU_tot_) using four subpopulations as shown in equation (1).

${CFU}_{tot}={CFU}_{RS,1}+{CFU}_{RS,2}+{CFU}_{SR,1}+{CFU}_{SR,2}+{CFU}_{SS,1}+{CFU}_{SS,2}+{CFU}_{RR,1}+{CFU}_{RR,2}$ (1)

where CFU_RS_ is the subpopulation resistant to COL and susceptible to CAZ, CFU_SR_ is the subpopulation susceptible to COL and resistant to CAZ, CFU_SS_ is the subpopulation susceptible to both COL and CAZ, and CFU_RR_ is the subpopulation resistant to both COL and CAZ. The sum of each state, 1 and 2, contributes to the total bacterial load.

The differential equations for the two states used to describe bacteria in state 1 and 2 for bacterial subpopulation (CFU_SR_) incorporating bacterial killing by COL and CAZ are shown in Equations (2) and (3).

$\frac{d\left( {CFU}_{SR,1} \right)}{dt}=REP\cdot k_{21}\cdot{CFU}_{SR,2}-k_{12,RS}\cdot{CFU}_{SR,1}-\left( {{Kill}_{COL,S}+Kill}_{CAZ,R} \right)\cdot{CFU}_{SR,1}$ (2)

$\frac{d\left( {CFU}_{SR,2} \right)}{dt}=-k_{21}\cdot{CFU}_{SR,2}+k_{12,SR}\cdot{CFU}_{SR,1}-\left( {{Kill}_{COL,S}+Kill}_{CAZ,R} \right)\cdot{CFU}_{SR,2}$ (3)

where REP is defined as the replication factor, and $2\cdot\left( 1-\frac{{CFU}_{tot}}{{CFU}_{MAX}+{CFU}_{tot}} \right)$. CFU_MAX_ is the maximum size of the bacterial population. The factor, 2, represents the doubling of bacteria during replication. The inverse of the mean replication time from state 2 to state 1, k_21_, was fixed to 50 h^-1^ for all subpopulations, while the inverse of the mean replication time from state 1 to state 2, k_12_, was estimated for each subpopulation (31). Kill_COL,S_ is the killing rate by COL for COL-susceptible subpopulations and is described by a second-order killing rate constant for the COL-susceptible subpopulation, K2S, and the assumed effective COL concentration at the target site, COL_EFF_. COL_EFF_ is a function of the COL concentration in the broth and the fractional occupancy of the COL-targeted receptor by Mg^2+^ and Ca^2+^ previously described (29). Kill_CAZ,R_ is the killing rate by CAZ for CAZ-resistant subpopulations and is described by a Hill-type equation following delay described using two transit compartments (Equation 4) representative of the inhibition of cell-wall synthesis during bacterial replication caused by CAZ inhibition of penicillin-binding proteins.

${Kill}_{CAZ,R}=\frac{K_{MAX,CAZ,R}\cdot{CAZ}^{\gamma_{CAZ}}}{\left( {KC}_{50,CAZ}\cdot OM\_effect \right)^{\gamma_{CAZ}}+{CAZ}^{\gamma_{CAZ}}}$ (4)

K_MAX,CAZ,R_ is the maximum killing rate by CAZ against the CAZ-susceptible subpopulations, KC_50,CAZ_ (referenced as KC_50_ in the RF model) is the CAZ concentration causing 50% of maximal killing, and $\gamma_{CAZ}$ is a Hill coefficient describing the CAZ killing effect. Similar equations were used to describe the bacterial subpopulations CFU_SR_, CFU_SS_, and CFU_RR_. The maximal killing effect of each subpopulation was described by differing killing rate parameters (K2S and K2R) for each subpopulation.

Overall, 21 system parameters were used to describe the MBM based on approximately 161 data points (7 sampling time points, 6x COL, 4x CAZ/AVI and 12x COL+ CAZ/AVI concentrations in addition to growth control) to estimate these parameters for each isolate (>7 samples per parameter). The median %standard error is below 30% for all except one of the MBM parameters. This is considered highly acceptable, and indicates that the parameters could be estimated precisely overall, which would not have been the case if there was substantial overfitting (overparameterization) (36).

*Subpopulation synergy*

We considered subpopulation synergy (i.e., colistin killing bacteria resistant to ceftazidime/avibactam and vice versa) in the model to describe the time course of bacterial killing and prevention of regrowth (34).

*Mechanistic synergy*

Mechanistic synergy was defined by Bliss independence in our quantification of antibiotic activity and is described by the disruption of the bacterial outer membrane by COL resulting in increased penetration and increased action of CAZ on penicillin-binding proteins (37). Mechanistic synergy was expressed as a decrease in CAZ concentration causing 50% killing (KC_50,CAZ_) of the CAZ-resistant, COL-susceptible subpopulation with increasing COL concentrations. This mechanistic synergy was incorporated in the model as the effect on the outer membrane (OM-effect) resulting in increased CAZ killing is described by equation (5) (38).

$OM\_effect=1-\left( \frac{I_{max,OM,COL}\cdot{C_{COL,EFF}}^{SYN}}{{C_{COL,EFF}}^{SYN}+{{IC}_{50,OM,COL}}^{SYN}} \right)$ (5)

where C_COL,EFF_ is the effective COL concentration described above, I_MAX,OM,COL_ is the maximum fractional decrease of KC_50,CAZ_ due to the effect of COL via outer membrane disruption, IC_50,OM,COL_ is the COL concentration that causes 50% of I_MAX,OM,COL_, and SYN is the Hill coefficient of the effect. This effect was applied to the subpopulation that was susceptible to COL but resistant to CAZ. Thus, if the MBM synergy term for a given isolate (I_MAX_) is > 0, then COL and CAZ/AVI demonstrate synergistic activity against the isolate under the Bliss independence definition.

*Initial conditions*

We estimated the total initial inoculum (log_10_CFU_0_) and the log-transformed mutation frequencies for the CFU_RS_, CFU_SR_, and CFU_RR_ subpopulations. Initial conditions were implemented as described previously (34, 39, 40).

*Error model*

The log_10_ viable counts were fit using an additive residual error model. For observations below the lower limit of quantification (100 CFU/mL), a previously developed residual error model was used to fit the number of colonies per plate (29). A proportional error model was used for observed colony counts with the same variance as the additive error on the log10 scale.

*Estimation*

Parameters were estimated using the importance sampling algorithm (pmethod=4) in S-ADAPT (version 1.57) (41) facilitated by SADAPT-TRAN (42). Models were evaluated by diagnostic plots, biological feasibility of parameter estimates, and objective function values.

*Random Forest (RF) algorithm*

To identify the clinical, genomic, and drug variables responsible for BSI mortality, a supervised classification RF model was built using the R packages caret (43) and randomForest (44). The primary clinical outcome for the model was 30-day mortality (either the patient died or was discharge to hospice care within 30 days from index CR*Kp* blood culture). We included baseline clinical characteristics and excluded treatment type as selection of treatment was at the discretion of the physician. Overall, 0.3% of data points were missing. Missing data were imputed using the median for numeric variables and mode for categorical variables.

We selected RF over other ML approaches due to its ability to perform well compared to other ML models at handling high-dimensional data with a high ratio of features to samples (i.e. "large p, small n problem") (45-47). Multiple studies across clinical, genomic and behavioral disciplines have also applied ML and Random Forest models to small datasets with < 200 samples (48-54). We applied a nested cross-validation (nCV) RF model with 5-fold CV for both the Outer and Inner Loops for the primary and secondary RF models. A nCV approach enabled isolation of an independent validation dataset in the Outer Loop and allowed for feature selection and hyperparameter tuning during model training and validation using "unseen" data (54, 55). This strategy is particularly relevant for smaller datasets where holding out samples for validation is costly.

For the primary RF, genes determined to be within the same operon were manually combined into a single representative variable. To reduce additional collinearity between genes, a representative gene was selected for highly correlated genes (Phi coefficient, r >90%). Categorical clinical variables and genes were also excluded if they had <10% or >90% frequency heterogeneity. Recursive Feature Elimination (RFE) and tuning of mtry occurred for each model in the Inner Loop using training data. ntree was held constant at 500 trees. A grid search to identify optimal RFE features sizes and mtry values was performed. RF models were optimized for the highest Accuracy. Accuracy is the number of correct predictions out of the total number of predictions. Validation was performed using an independent dataset representing 20% of the total dataset. To control for instability in importance estimates only a consensus set of variables found within the majority ($\geq$10) of training models were ranked by importance. Prior and additional antibiotic exposure were not incorporated into the RF models. The top 10 most important variables were ranked by permutation feature importance and combined with the MBM variables into a second RF model for patients within the secondary cohort (n=22). Permutation feature importance is the decrease in model accuracy when a single feature/variable is randomly permutated.

For the secondary RF, variables with low heterogeneity were re-evaluated and excluded. Feature selection and parameter tuning were not performed in the secondary RF model due to its reduced size. Default values were used for both mtry (mtry=$\surd(Number of variables))$and ntree (ntree=500). In total, 15 variables were tested in the secondary RF model (2 clinical, 5 bacterial genetic, 6 MBM, and the absolute MIC values for COL and CAZ/AVI). In the secondary RF model, we observed minimal overfitting as the Accuracy achieved during training was 69.3% compared to 73.0% on the validation dataset.

**Supplementary Tables**

**Table S1.** Variables included in Random Forest models. Variables are grouped as either (1) clinical, (2) bacterial genetic, or (3) drug (susceptibilities and mechanism-based modeling (MBM)). Bolded variables were included in a second Random Forest model using only data from the secondary patient cohort and drug-based variables.

| **1: Clinical Variables** | | |
| --- | --- | --- |
| Age | | |
| Race | | |
| Sex | | |
| Highest Creatinine Levels | | |
| Highest Peripheral White Blood Cell Count | | |
| Highest Neutrophil Cell Count | | |
| Lowest Hemoglobin Levels | | |
| Highest Temperature | | |
| Immunocompromised | | |
| Cerebrovascular Disease | | |
| Chronic Kidney Disease | | |
| Cirrhosis | | |
| Congestive Heart Failure | | |
| Diabetes Mellitus | | |
| History of Coronary Artery Disease/Myocardial Infarction | | |
| History of Malignancy | | |
| **Hypotension** | | |
| Peripheral Vascular Disease | | |
| Renal Failure | | |
| Central Venous Line | | |
| **Mechanical Ventilation** | | |
| Surgery | | |
| **2: Bacterial Genetic Variables** | | **Protein Function** |
| **Antibiotic Resistance** | ***aac(3)-IV* (DQ241380)** | Aminoglycoside acetyltransferase |
|  | *aac(6')-Ib* (M21682) | Aminoglycoside acetyltransferase |
|  | ***aadA2* (AF156486)** | Aminoglycoside nucleotidyltransferase |
|  | *aph(3')-Ia* (BX664015) | Aminoglycoside phosphotransferase |
|  | *bla*_CTX-M-15_ (AY044436) | Extended-spectrum beta-lactamase |
|  | *bla*_OXA-1_ (AFB82783) | Beta-lactamase |
|  | *bla*_OXA-9_ (KQ089875) | Beta-lactamase |
|  | *bla*_SHV-12_ (KF976405) | Extended-spectrum Beta-lactamase |
|  | *bla*_SHV-182_ (KP050489) | Beta-lactamase |
|  | *bla*_TEM-1A_ (HM749966) | Beta-lactamase |
|  | *bla*_TEM-1B_ (AY458016) | Beta-lactamase |
|  | *bla*_TEM-122_ (AY307100) | Beta-lactamase |
|  | *bla*_TEM-150_ (AM183304) | Beta-lactamase |
|  | *catA1* (V00622) | Chloramphenicol acetyltransferase |
|  | ColRNAI_1 (DQ298019) | Plasmid replication |
|  | *dfrA12* (AM040708) | Dihydrofolate reductase |
|  | *dfrA14* (DQ388123) | Dihydrofolate reductase |
|  | IncFIB(K)_1_Kpn3 (JN233704) | Plasmid replication |
|  | IncFIB(pQil)_1_pQil (JN233705) | Plasmid replication |
|  | IncFII(Yp)_1_Yersenia (CP000670) | Plasmid replication |
|  | IncI2_1_Delta (AP002527) | Plasmid replication |
|  | **IncR_1 (DQ449578)** | Plasmid replication |
|  | IncX3_1 (JN247852) | Plasmid replication |
|  | *sul1* (EU780013) | Dihydropteroate synthase |
|  | ***sul3* (AJ459418)** | Dihydropteroate synthase |
|  | *tetA* (AF534183) | Tetracycline efflux pump |
|  | Tn*4401b* | Tn*3*-like transposon |
| **Adherence** | *fimE* (KPHS_43530) | Fimbria Regulatory Protein [Type 1 fimbriae] |
|  | *fimH* (KPR_1736) | Adhesin [Type 1 fimbriae] |
|  | *mrkJ* (KPHS_43420) | Transcriptional Regulator [Type 3 fimbriae] |
|  | *mrkF* (KPHS_43430) | Fimbrial anchor [Type 3 fimbriae] |
|  | *mrkD* (KPHS_43440) | Adhesin [Type 3 fimbriae] |
|  | *mrkC* (KPHS_43450) | Outer Membrane Usher [Type 3 fimbriae] |
|  | *mrkB* (KPHS_43460) | Protein Chaperone [Type 3 fimbriae] |
|  | *mrkH* (KP1_4551) | Transcriptional Regulator [Type 3 fimbriae] |
| **Iron**  **Acquisition** | *ybtS* (LR792628) | Yersiniabactin biosynthesis |
|  | *entB* (NP_752613) | Isochorismatase |
|  | *entF* (KPN_00605) | Enterobactin synthase subunit |
|  | *fepA* (KPHS_14370) | Ferrienterobactin receptor |
| **Capsule** | KPN_02514 | Putative acid phosphatase |
|  | KPHS_35550 | dTDP-4-dehydrorhamnose 35-epimerase |
|  | *manB* (KPR_1600) | Phosphomannomutase dehydrogenase |
|  | *rcsB* (KPN_02638) | Transcriptional regulator |
|  | *rfbA* (KPR_1604) | Integral membrane O-antigen translocator protein |
|  | KPN2242_15480 | Hypothetical protein |
|  | KPN2242_15485 | Hypothetical protein |
|  | *rfbD* (FO203501) | Probable UDP-galactopyranose mutase |
|  | **KPR_1609** | Galactosyl transferase |
| **Type VI**  **Secretion System** | *hcp*/*tssD* (KPHS_23020) | Type VI secretion system protein [T6SS-I] |
|  | *tli1* (KPHS_23060; KPHS_23070; KPHS_23080; KPN2242_09805) | Type VI secretion system immunity protein [T6SS-I] |
|  | *tssF* (KPN_01347) | Hypothetical protein [T6SS-I] |
|  | *dotU* (A79E_1966) | Transmembrane protein [T6SS-III] |
|  | *impK* (N559_2001) | Type VI secretion system protein [T6SS-III] |
|  | *icmF* (KPHS_32500) | Hypothetical protein [T6SS-III] |
|  | *rbsB* (A79E_1989) | Ribose ABC transport system periplasmic ribose-binding protein [T6SS-III] |
|  | KPN_02244 | Hypothetical protein [T6SS-III] |
| **3: Drug Variables: Susceptibilities and Mechanism-Based Modeling** | | |
| **MIC** | **MIC COL** | MIC of colistin |
|  | **MIC CAZ/AVI** | MIC of ceftazidime/avibactam |
| **MBM** | **EC_50_** | Fraction of receptors unoccupied by Ca^2+^ and Mg^2+^, resulting in 50% COL concentration at the outer membrane target site relative to COL concentration in broth |
|  | **IC_50_** | COL concentration causing 50% of I_MAX,OM_ |
|  | **I_MAX,OM_** | Maximum fractional decrease of KC_50_ by COL via outer membrane disruption |
|  | **KC_50_** | CAZ concentration needed to achieve 50% of maximum killing |
|  | **K_MAX,R_** | Maximum killing rate constant for CAZ in CAZ^R^ population |
|  | **K_MAX,S_** | Maximum killing rate constant for CAZ in CAZ^S^ population |

MIC, minimum inhibitory concentration; MBM, mechanism-based model

**Table S2.** Treatment characteristics of primary cohort (n=49)

| **Treatment Characteristics** | **Patients, No (%)** | |
| --- | --- | --- |
| Additional antibiotics | Prior to index culture**^a^** | On or after index culture**^b^** |
| Carbapenem | 11 (22) | 28 (57) |
| Aminoglycoside | 5 (10) | 28 (57) |
| Tigecycline | 4 (8) | 27 (55) |
| Vancomycin | 18 (37) | 20 (41) |
| Piperacillin/tazobactam | 9 (18) | 13 (27) |
| Quinolone | 12 (24) | 10 (20) |
| Daptomycin | 1 (2) | 10 (20) |
| Trimethoprim/sulfamethoxazole | 7 (14) | 10 (20) |
| Cephalosporin | 5 (10) | 6 (12) |
| Metronidazole | 6 (12) | 2 (4) |
| Linezolid | 2 (4) | 2 (4) |
| Aztreonam | 0 | 1 (2) |
| Rifaximin | 0 | 1 (2) |
| Unknown | 5 (10) | 1 (2) |
| Doxycycline | 1 (2) | 0 |

**^a^**Antibiotics were administered within 14 days prior to index blood CR*Kp* culture

**^b^**Antibiotics were administered on date of index blood CR*Kp* culture or up to 10 days after the index culture

**Table S3.** Baseline clinical characteristics of secondary patient cohort^a^

| **Characteristic, n (% or IQR)** | **Alive**  **(n=10)** | **Deceased**  **(n=12)** | **Total**  **(n=22)** |
| --- | --- | --- | --- |
| Sex; female | 3 (30) | 7 (58) | 10 (46) |
| Race |  |  |  |
| Caucasian | 6 (60) | 8 (67) | 14 (64) |
| African American | 2 (20) | 4 (33) | 6 (27) |
| Other | 2 (20) | 0 (0) | 2 (9) |
| Ethnicity |  |  |  |
| Hispanic or Latino | 0 (0) | 0 (0) | 0 (0) |
| Age (yrs) | 57 (47, 70) | 69 (63, 76) | 68 (57, 74) |
| Highest creatinine level (mg/dL)**^b^** | 1.0 (1.0, 2.0) | 2.0 (1.0, 3.0) | 1.5 (1.0, 2.8) |
| Highest neutrophil count (1,000 cells/μL)**^b^** | 12 (2.5, 17) | 13 (11, 17) | 13 (10, 17) |
| Lowest hemoglobin level (g/dL)**^b^** | 8.5 (7.3, 10) | 9.0 (8.0, 10) | 9.0 (8.0, 10) |
| Highest peripheral white blood cell count (1000 cells/μL)**^b^** | 14 (3.5, 21) | 18 (12, 28) | 17 (11, 26) |
| Highest temperature (°C)**^b^** | 38.2 (36.9, 39.2) | 37.5 (37.1, 38.0) | 37.7 (36.8, 38.7) |
| Immunocompromised | 4 (44) | 3 (30) | 7 (37) |
| Congestive heart failure | 1 (10) | 7 (64) | 8 (38) |
| Peripheral vascular disease | 1 (10) | 2 (18) | 3 (14) |
| Cerebrovascular disease | 1 (10) | 2 (18) | 3 (14) |
| Diabetes mellitus | 6 (60) | 5 (46) | 11 (52) |
| Malignancy within last 5 yrs | 3 (30) | 3 (27) | 6 (29) |
| Chronic kidney disease | 4 (40) | 7 (64) | 11 (52) |
| Cirrhosis | 1 (10) | 2 (18) | 3 (14) |
| Renal failure | 7 (70) | 10 (83) | 17 (77) |
| Pitt bacteremia score | 3 (2, 4) | 4 (4, 4) | 4 (3, 4) |
| Charlson comorbidity index | 3 (3, 4) | 6 (4, 6) | 4 (3, 6) |
| History of coronary artery disease/myocardial infarction | 2 (20) | 2 (18) | 4 (19) |
| Hypotension | 5 (50) | 11 (92) | 16 (73) |
| Surgery | 5 (50) | 4 (33) | 9 (41) |
| Central venous line | 8 (89) | 11 (92) | 19 (91) |
| Mechanical ventilation | 7 (70) | 10 (83) | 17 (77) |
| Treatment |  |  |  |
| Monotherapy COL | 3 (30) | 11 (92) | 14 (64) |
| Monotherapy CAZ/AVI | 3 (30) | 1 (8) | 4 (18) |
| Combination therapy | 4 (40) | 0 (0) | 4 (18) |
| Time to CAZ/AVI treatment (days)**^c^** | 3 (2, 4) | 0 (0, 0) | 3 (1, 4) |
| Time to COL treatment (days)**^c^** | 3 (2, 4) | 2 (1, 3) | 2 (1, 4) |
| Time from admission to index culture (days) | 0 (0, 18) | 3 (0, 18) | 1 (0, 18) |

**^a^** IQR, interquartile range; COL, colistin; CAZ/AVI ceftazidime/avibactam.

**^b^** Recorded on date of index CR*Kp* blood culture

**^c^** For patients receiving mono- or combination therapy, time to treatment of individual drugs from index CR*Kp* blood culture

**Table S4.** Baseline characteristics of isolates from the secondary patient cohort^a^

| **Characteristic, n (%)** | **Alive**  **(n=10)** | | **Deceased**  **(n=12)** | **Total**  **(n=22)** | |
| --- | --- | --- | --- | --- | --- |
| Strain Type |  | |  |  | |
| 258 | 8 (80) | | 10 (83) | 18 (82) | |
| 11 | 0 | | 1 (8) | 1 (5) | |
| Other**^b^** | 2 (20) | | 1 (8) | 3 (14) | |
| *wzi* capsule type |  | |  |  | |
| 154 | 2 (20) | | 6 (50) | 8 (36) | |
| 29 | 6 (60) | | 5 (42) | 11 (50) | |
| 27 | 0 | | 1 (8) | 1 (5) | |
| Other | 2 (20) | | 0 | 2 (9) | |
| *β*-lactamases**^c^** | |  |  | |  |
| Carbapenemase |  | |  |  | |
| *bla*_KPC-2_ | 7 (70) | | 6 (50) | 13 (59) | |
| *bla*_KPC-3_ | 3 (30) | | 6 (50) | 9 (41) | |
| *bla*_OXA-232_ | 1 (10) | | 0 | 1 (5) | |
| ESBL |  | |  |  | |
| *bla*_SHV-12_ | 6 (60) | | 9 (75) | 15 (68) | |
| *bla*_CTX-M_**^d^** | 2 (20) | | 1 (8) | 3 (14) | |
| Antimicrobial non-susceptibility**^e^** |  | |  |  | |
| COL | 1/10 (10) | | 1/12 (8) | 2/22 (9) | |
| CAZ/AVI | 0/10 | | 0/12 | 0/22 | |

^a^ ESBL, extended spectrum β-lactamase; COL, colistin; CAZ/AVI, ceftazidime/avibactam

^b^ Includes ST231(1), ST418(1), and ST76(1)

^c^ Isolates could carry multiple genes encoding β-lactamases

^d^ Includes *bla*_CTX-M-14_ (1) and *bla*_CTX-M-15_ (2)

^e^ Includes only those isolates tested for susceptibilities

**Table S5.** Final estimates of parameters included in the mechanism-based model for the secondary cohort (n=22) of CR*Kp* isolates. The extent of precision within each parameter is represented by the coefficient of variation (CV%).^a^

| **Parameter** | **Description** | **Final Estimate**  **(Median [Range])** | **CV%**  **(Median [Range])** |
| --- | --- | --- | --- |
| LogCFU_0_  (log_10_ CFU/ml) | Initial inoculum | 6.17 [5.64 – 6.30] | 0 [0 – 4.64]^b^ |
| LogCFU_max_  (log_10_ CFU/ml) | Maximum carrying capacity | 9.36 [9.04 – 10.0] | 0 [0 – 11.8]^b^ |
| Log_MF_RS_ | Log_10_mutation frequency of COL^R^ and CAZ^S^ subpopulation | -4.57 [-1.72 – -22.2] | 15.1 [6.85 – 60.4] |
| Log_MF_SR_ | Log_10_mutation frequency of COL^S^ and CAZ^R^ subpopulation | -4.05 [-0.270 – -11.7] | 10.1 [3.86 – 31.1] |
| Log_MF_RR_ | Log_10_mutation frequency of COL^R^ and CAZ^R^ subpopulation | -5.91 [-2.39 – -18.5] | 7.94 [3.78 – 31.5] |
| k_21_ (h^-1^) | Replication rate constant | 50 fixed | fixed |
| MTT_12,SS_(min) | Mean transit time for COL^S^ and CAZ^S^ subpopulation | 68.7 [48.3 – 180] | 13.5 [5.90 – 34.2] |
| MTT_12,SR_ (min) | Mean transit time for COL^S^ and CAZ^R^ subpopulation | 151 [46.2 – 508] | 10.4 [6.17 – 40.4] |
| MTT_12,RS_ (min) | Mean transit time for COL^R^ and CAZ^S^ subpopulation | 48.0 [17.7 – 1273] | 9.39 [4.15 – 36.8] |
| MTT_12,RR_ (min) | Mean transit time for COL^R^ and CAZ^R^ subpopulation | 110 [20.5 – 918] | 9.35 [5.05 – 170] |
| K_2S_ [L/(mg*h)] | Second-order killing rate constant for COL in COL^S^ subpopulations | 10.4 [0.446 – 133] | 16.7 [5.83 – 33.1] |
| K_2R_ [L/(mg*h)] | Second-order killing rate constant for COL in COL^R^ subpopulations | 0.130 [0.005 – 0.657] | 25.6 [7.52 – 89.6] |
| EC_50_ | Fraction of receptors unoccupied by Ca^2+^ and Mg^2+^, resulting in 50% COL concentration at the outer membrane target site relative to COL concentration in broth | 0.417 [0.218 – 25.4] | 27.0 [16.3 – 136] |
| H_COL_ | Hill coefficient of COL receptor occupancy | 0.219 [0.086 – 2.39] | 27.1 [6.31 – 91.4] |
| K_MAX,S_ (h^-1^) | Maximum killing rate constant for CAZ in CAZ^S^ subpopulations | 12.8 [6.41 – 52.6] | 14.6 [4.94 – 40.6] |
| K_MAX,R_ (h^-1^) | Maximum killing rate constant for CAZ in CAZ^R^ subpopulations | 1.36 [0.007 – 13.5] | 26.8 [12.4 – 59.0] |
| TR (h) | Time delay of CAZ effect through each transit compartment | 0.214 [0.051 – 0.862] | 20.5 [11.6 – 56.0] |
| KC_50_ (mg/L) | CAZ concentration needed to achieve 50% of maximum killing | 0.935 [0.492 – 2.06] | 24.5 [6.20 – 84.5] |
| H_CAZ_ | Hill coefficient of CAZ killing effect | 0.837 [0.461 – 1.80] | 26.0 [12.6 – 63.0] |
| I_MAX,OM_ | Maximum fractional decrease of KC_50_ by COL via outer membrane disruption | 0.603 [-0.264 – 0.725] | See Table S6 for 95% CI^c^ |
| IC_50_ (mg/L) | COL concentration causing 50% of I_MAX,OM_ | 1.02 [0.860 – 1.41] | 25.6 [13.5 – 71.0] |
| H_SYN_ | Hill coefficient of COL effect on outer membrane | 0.993 [0.680 – 1.87] | 28.1 [13.1 – 121] |
| CV (%) | Coefficient of variation | 51.4 [22.5 – 75.3] | 10.5 [7.10 – 29.3] |

^a^ R, resistant; S, sensitive

^b^ CV% of 0 indicates the parameter was fixed for the specific isolate

^c^ The 95% CI is provided as this parameter is logistically transformed in the model.

**Table S6.** I_MAX,OM_ parameter estimates for each isolate

| **Isolate** | **Estimate** | **95% Confidence Interval** |
| --- | --- | --- |
| CRK0088 | 0.633 | (0.222 - 0.913) |
| CRK0089 | 0.649 | (0.427 - 0.821) |
| CRK0091 | 0.654 | (0.535 - 0.757) |
| CRK0094 | 0.644 | (0.201 - 0.928) |
| CRK0098 | 0.625 | (0.409 - 0.8) |
| CRK0064 | 0.654 | (0.387 - 0.849) |
| CRK0042 | -0.012 | (-0.32 - 0.296) |
| CRK0078 | 0.637 | (0.423 - 0.808) |
| CRK0079 | 0.652 | (0.042 - 0.988) |
| CRK0084 | 0.654 | (0.26 - 0.91) |
| CRK0044 | -0.264 | (-0.734 - 0.206) |
| CRK0012 | 0.652 | (0.142 - 0.955) |
| CRK0022 | 0.644 | (0.018 - 0.994) |
| CRK0030 | 0.674 | (0.404 - 0.863) |
| CRK0031 | -0.168 | (-0.717 - 0.382) |
| CRK0033 | 0.658 | (0.314 - 0.89) |
| CRK0391 | -0.052 | (-0.701 - 0.598) |
| CRK0392 | 0.658 | (0.156 - 0.953) |
| CRK0394 | -0.258 | (-0.612 - 0.096) |
| CRK0396 | 0.650 | (0.288 - 0.895) |
| CRK0393 | 0.656 | (0.271 - 0.907) |
| CRK0395 | -0.001 | (-0.272 - 0.27) |

**Table S7.** Distribution of the top genetic variables identified by the primary RF models in CR*Kp* isolated from the primary and secondary patient cohort.

|  | **Primary Patient Cohort** | | | **Secondary Patient Cohort** | | |
| --- | --- | --- | --- | --- | --- | --- |
| **Gene, n (%)** | **Total**  **(n=49)** | **Alive**  **(n=33)** | **Died**  **(n=16)** | **Total**  **(n=22)** | **Alive**  **(n=10)** | **Died**  **(n=12)** |
| ***aac(3)-IV*** | 13/49 (27) | 6/33 (18) | 7/16 (44) | 7/22 (32) | 1/10 (10) | 6/12 (50) |
| ***aadA2*** | 32/49 (65) | 18/33 (55) | 14/16 (88) | 16/22 (73) | 5/10 (50) | 11/12 (92) |
| **IncR** | 34/49 (69) | 19/33 (58) | 15/16 (94) | 17/22 (77) | 5/10 (50) | 12/12 (100) |
| **KPN2242_15480^a^** | 39/49 (80) | 24/33 (73) | 15/16 (94) | 20/22 (91) | 8/10 (80) | 12/12 (100) |
| **KPR_1609** | 23/49 (47) | 13/33 (39) | 10/16 (63) | 9/22 (41) | 2/10 (20) | 7/12 (58) |
| ***mrkC*^a^** | 40/49 (82) | 24/33 (73) | 16/16 (100) | 21/22 (95) | 9/10 (90) | 12/12 (100) |
| ***sul3*** | 17/49 (35) | 7/33 (21) | 10/16 (63) | 8/22 (36) | 1/10 (10) | 7/12 (58) |

^a^ Excluded from the secondary RF model due to low heterogeneity

**Supplementary Figures**

**
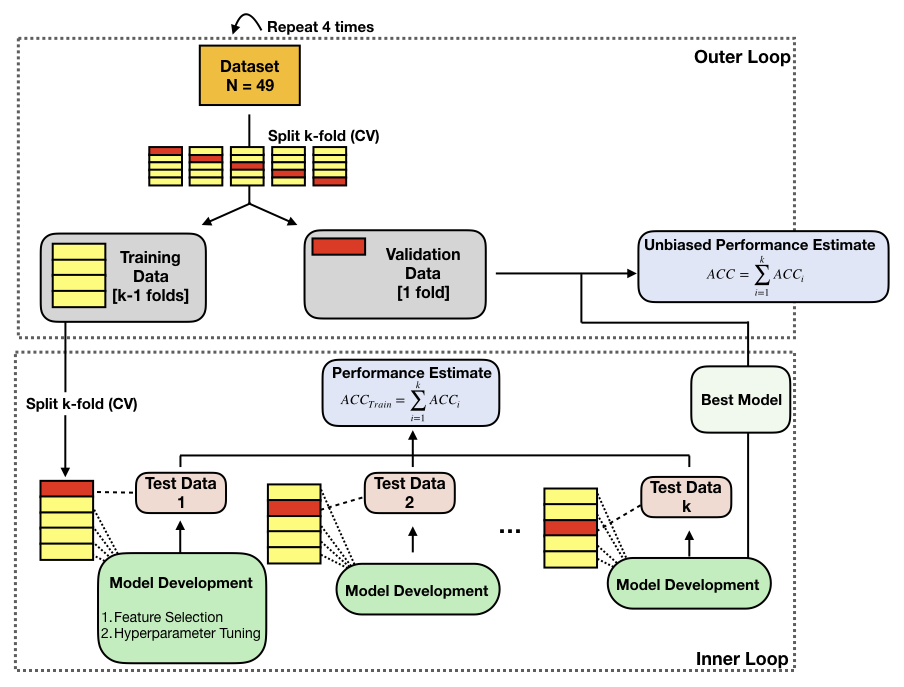
**

**Supplemental Figure S1.** Schematic of nested cross-validation approach used to generate the primary and secondary random forest models. CV: cross-validation; ACC: accuracy

**
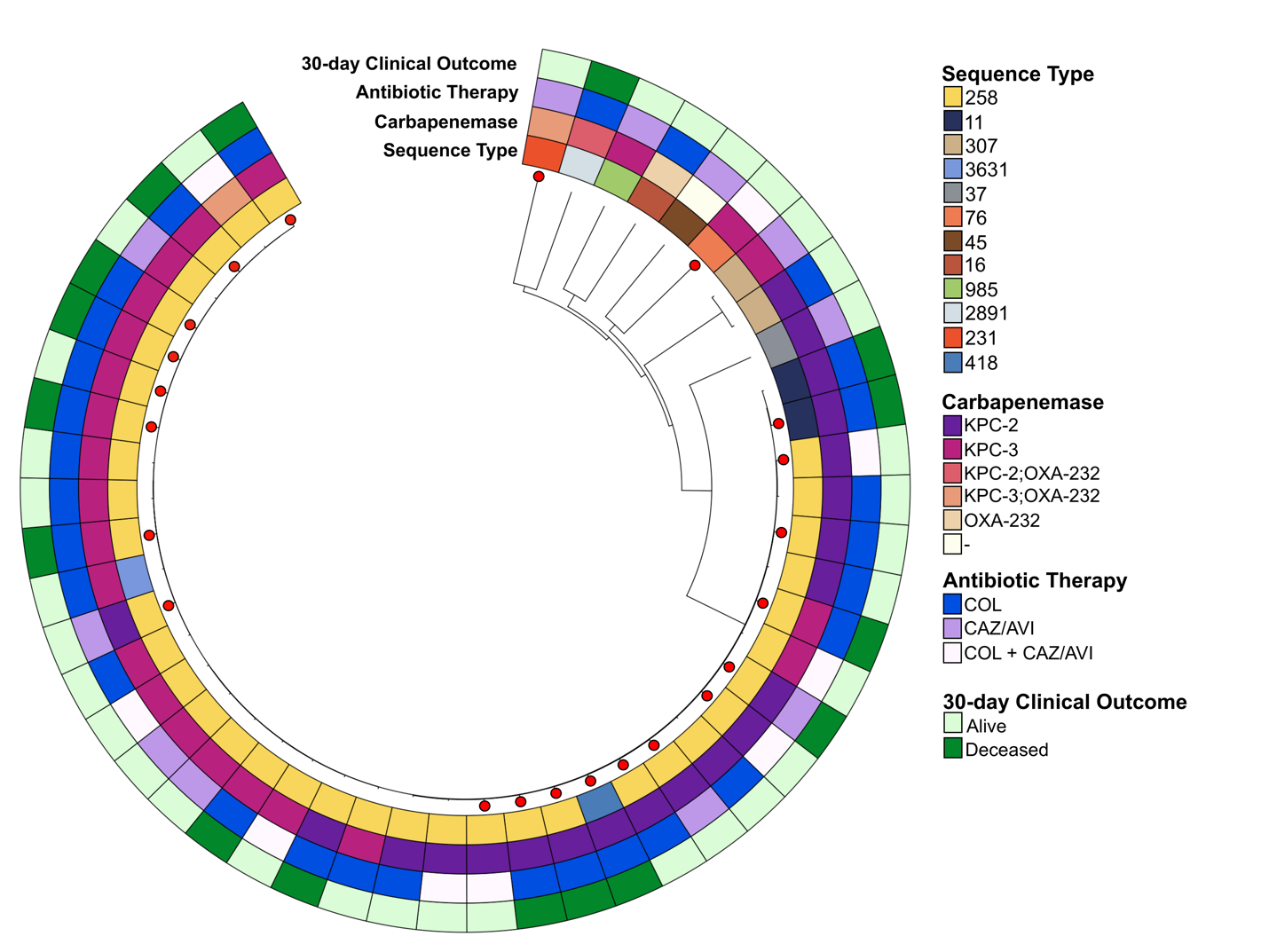
**

**Supplemental Figure S2.** Genetic relationships between CR*Kp* isolates based on a maximum likelihood tree analysis. Bacterial isolates from patients within the secondary cohort (n=22) are indicated by an orange circle at the tip. Metadata for each isolate are aligned within concentric rings. COL: colistin, CAZ: ceftazidime, AVI: avibactam

**
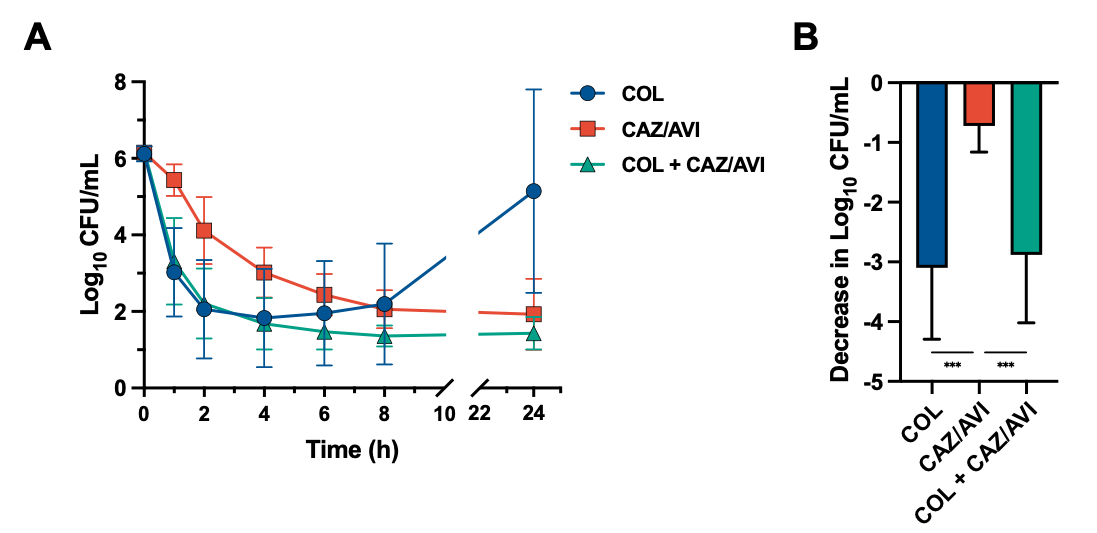
**

**Supplemental Figure S3.** (A) The average log_10_ CFU/mL for all CR*Kp* isolates (n=22) evaluated via SCTK assays over 24 h under the maximum static drug concentrations, and (B) the extent of initial bacterial killing measured as the average decrease in log_10_ CFU/mL within 1 h following exposure of bacteria to COL, CAZ/AVI or COL+CAZ/AVI. Drug concentrations considered to develop this figure and perform the analysis: COL: 16 mg/L; CAZ/AVI: 128/32 mg/L; COL+CAZ/AVI: 4 mg/L + 64/16 mg/L. Error bars represent mean $\pm$ SD. ****P*<0.0001 compared to log_10_ CFU/mL at time 0 by Kruskal-Wallis test with secondary testing using Dunn's multiple comparisons test.

***
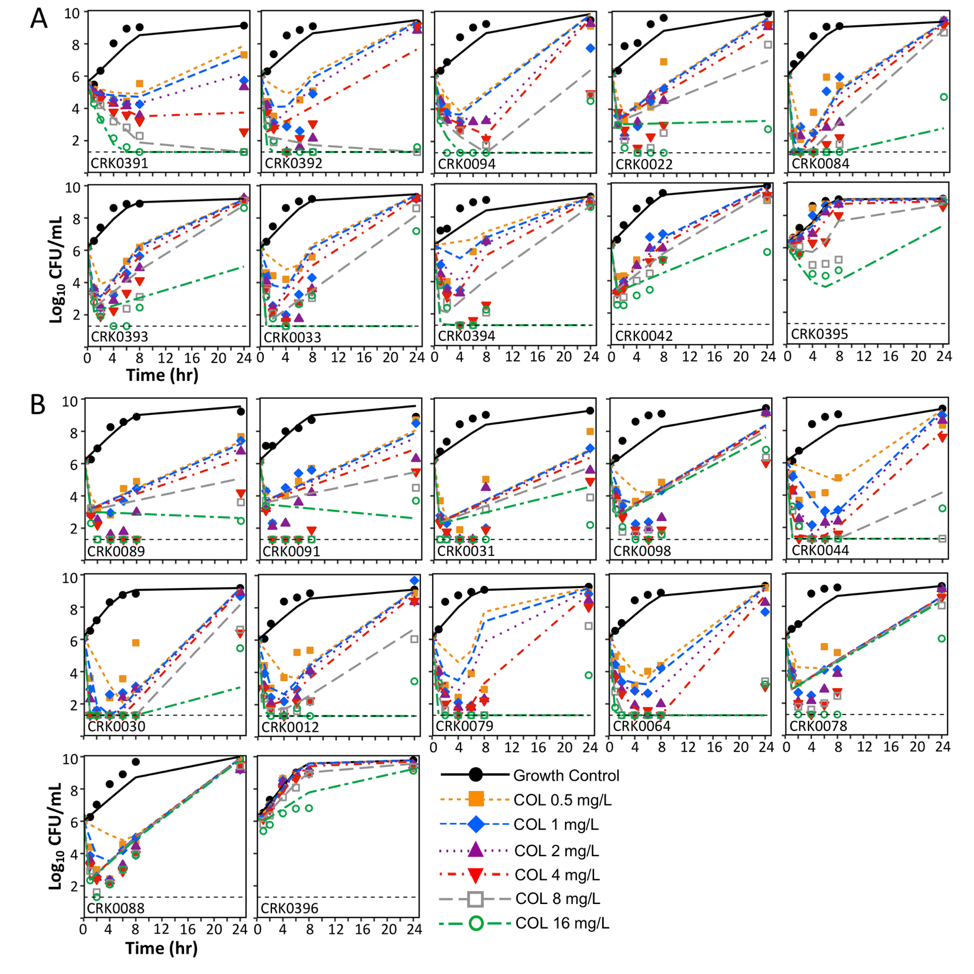
***

**Supplemental Figure S4.** The rate and magnitude of bacterial killing were characterized with a range of static COL concentrations over 24 h. Each panel summarizes observed (symbols) colony forming units (CFU) for each isolate. Model fits are shown as lines. A growth control was cultured without antibiotics. Isolates are grouped by isolation from patients who were either **(A)** alive or **(B)** deceased within 30 days from index CR*Kp* blood culture. Bacterial growth was measured as log_10_ CFU/mL from plated bacterial samples.


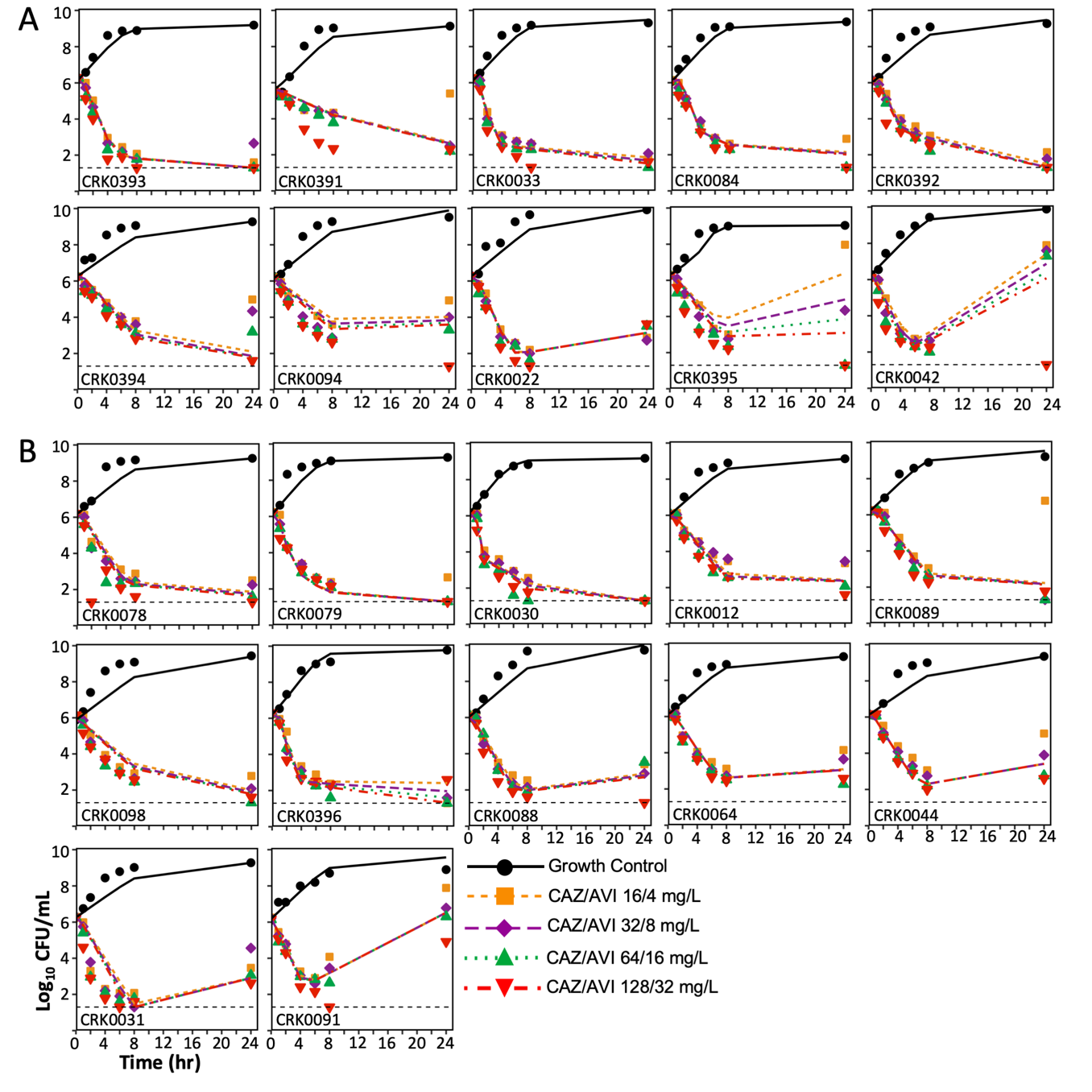


**Supplemental Figure S5.** The rate and magnitude of bacterial killing were characterized with a range of static CAZ/AVI concentrations over 24 h. Each panel summarizes observed (symbols) colony forming units (CFU) for each isolate. Model fits are shown as lines. A growth control was cultured without antibiotics. Isolates are grouped by isolation from patients who were either **(A)** alive or **(B)** deceased within 30 days from index CR*Kp* blood culture. Bacterial growth was measured as log_10_ CFU/mL from plated bacterial samples.

**
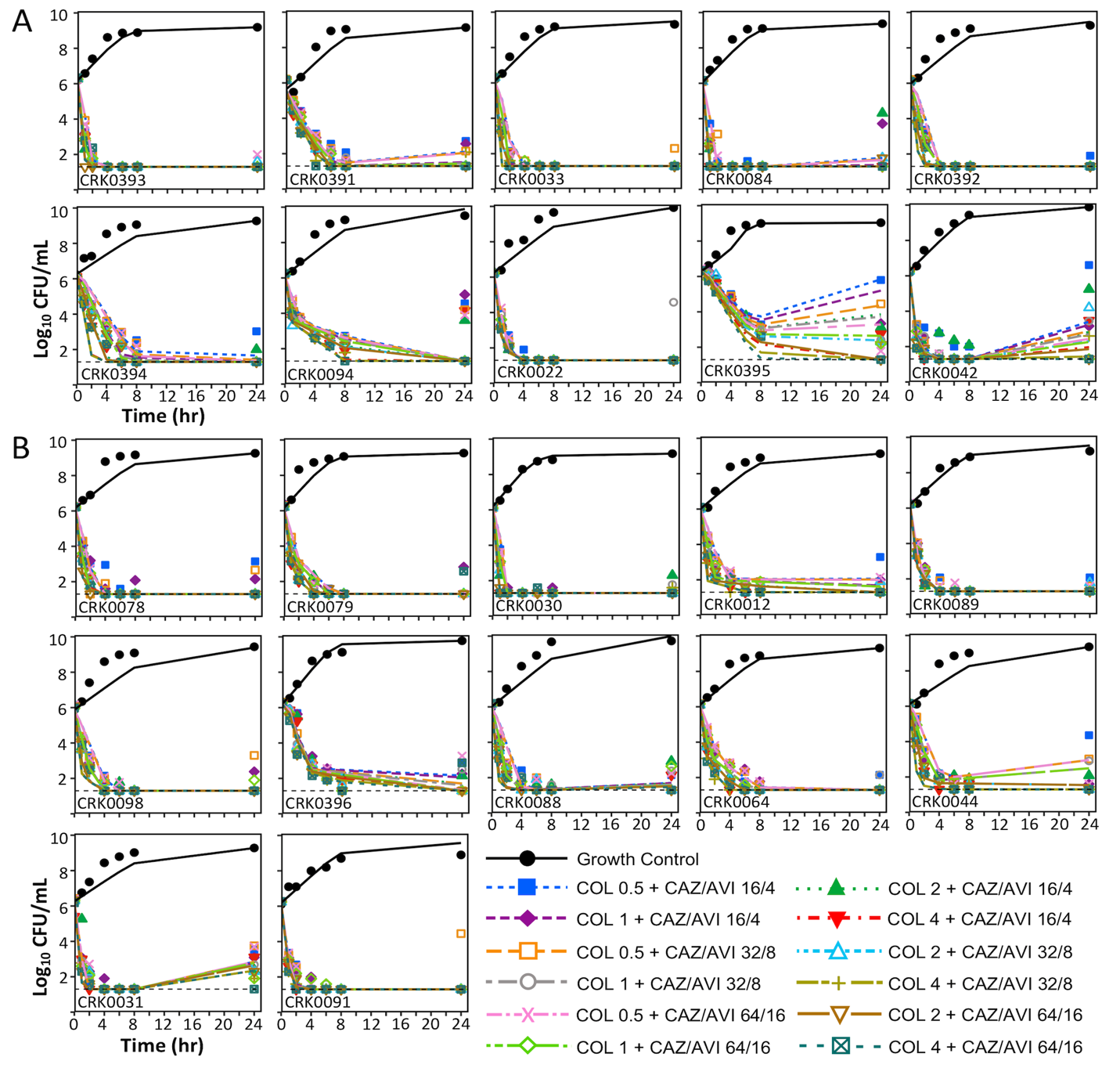
**

**Supplemental Figure S6.** The rate and magnitude of bacterial killing characterized for a range of static COL (mg/L) and CAZ/AVI (mg/L) concentrations over 24 h. Each panel summarizes observed (symbols) colony forming units (CFU) for each isolate. Model fits are shown as lines. A growth control was cultured without drug. Isolates are grouped by isolation from patients who were either **(A)** alive or **(B)** deceased within 30 days from index CR*Kp* blood culture. Bacterial growth was measured as log_10_ CFU/mL from plated bacterial samples.


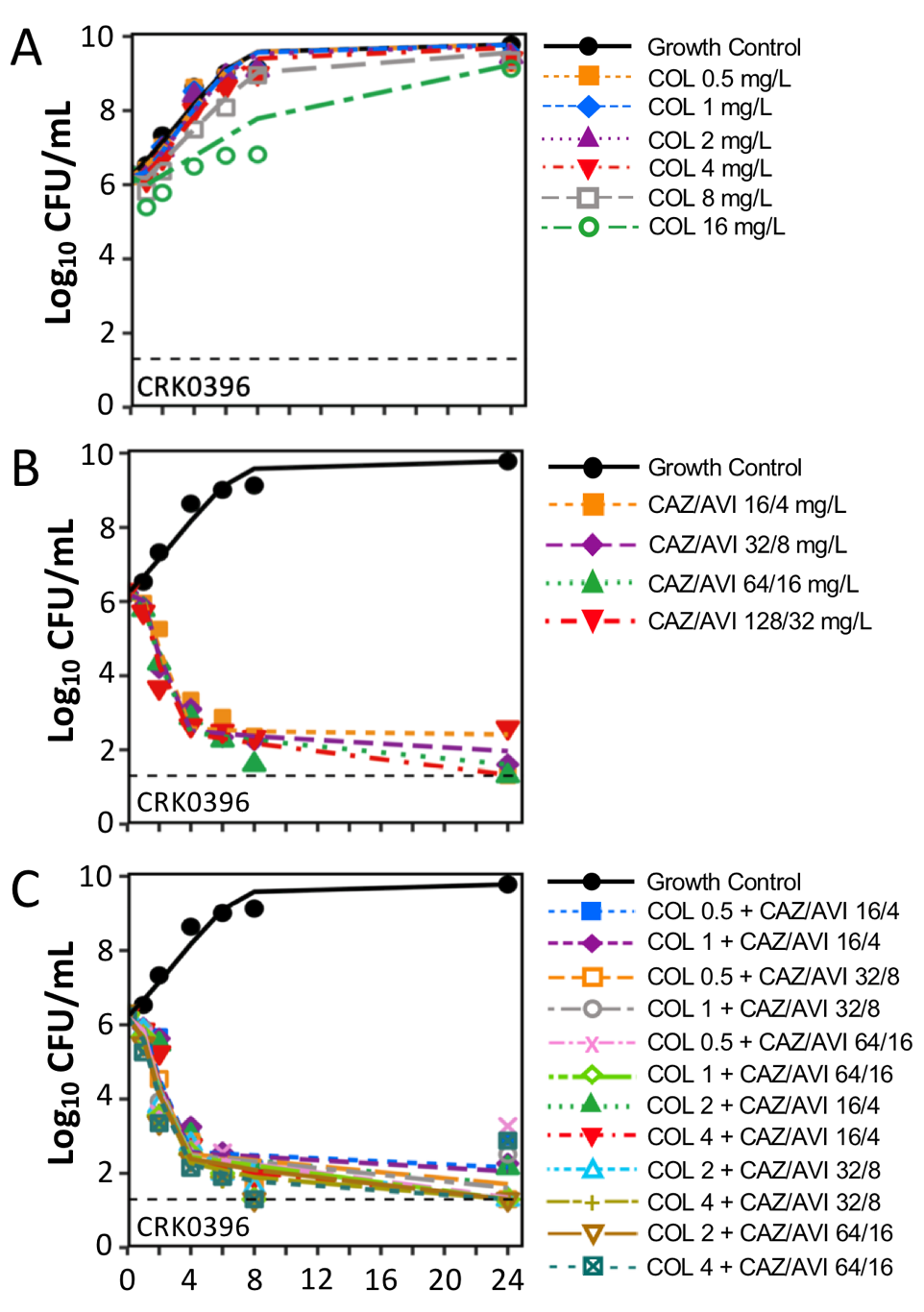


**Supplemental Figure S7.** The rate and magnitude of bacterial killing characterized for a range of static COL (mg/L) **(A)**, CAZ/AVI (mg/L) **(B)**, and COL+CAZ/AVI **(C)** concentrations over 24 h. Each panel summarizes observed (symbols) colony forming units (CFU) per mL for isolate CRK0396. Model fits are shown as lines. A growth control was cultured without drug. Bacterial growth was measured as log_10_ CFU/mL from plated bacterial samples.

**
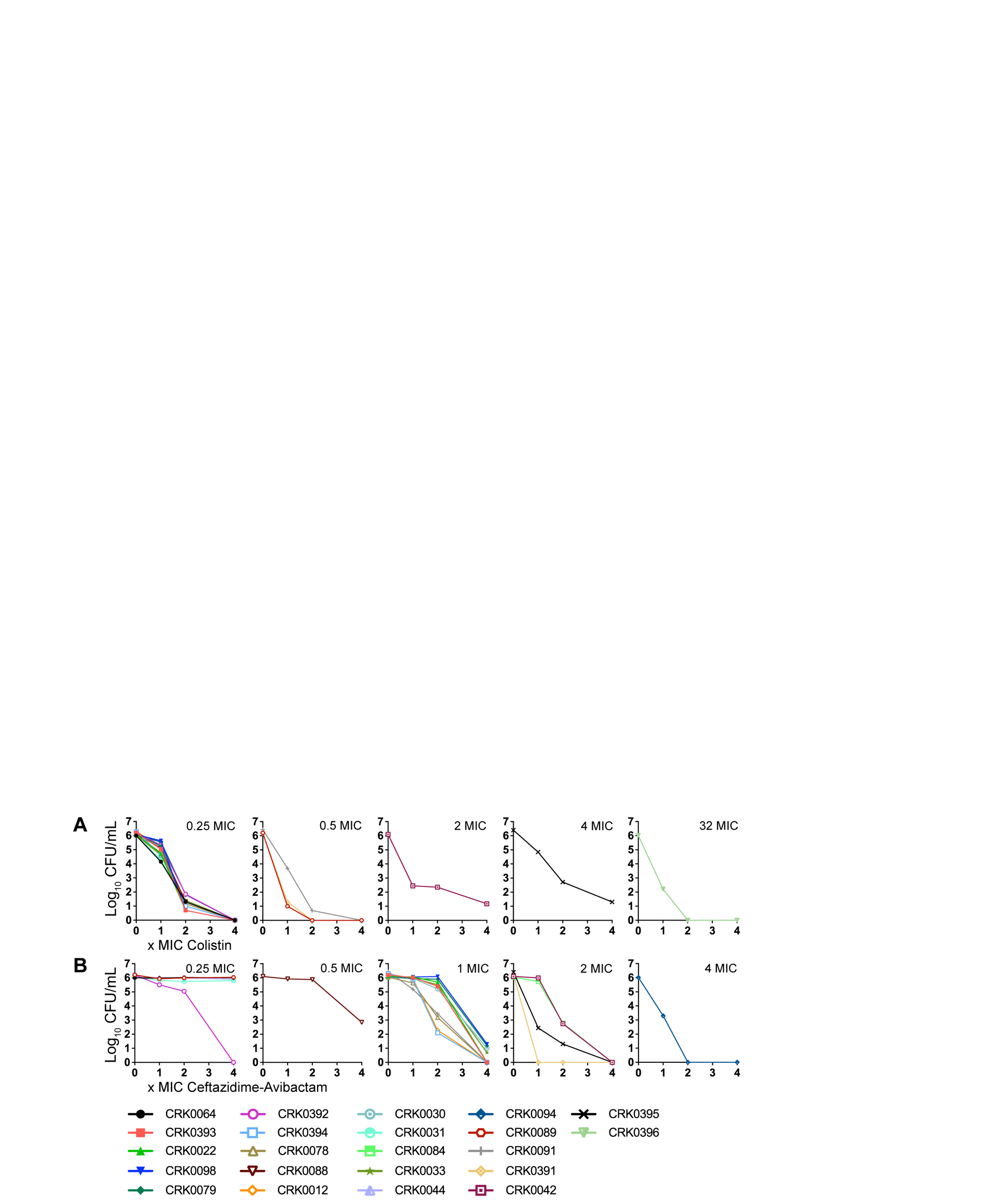
**

**Supplemental Figure S8.** Population analysis profiles at baseline showing the heterogeneity in susceptibility for 22 CR*Kp* isolates to either (A) colistin or (B) ceftazidime-avibactam. Isolates are grouped based on their MIC to each drug.


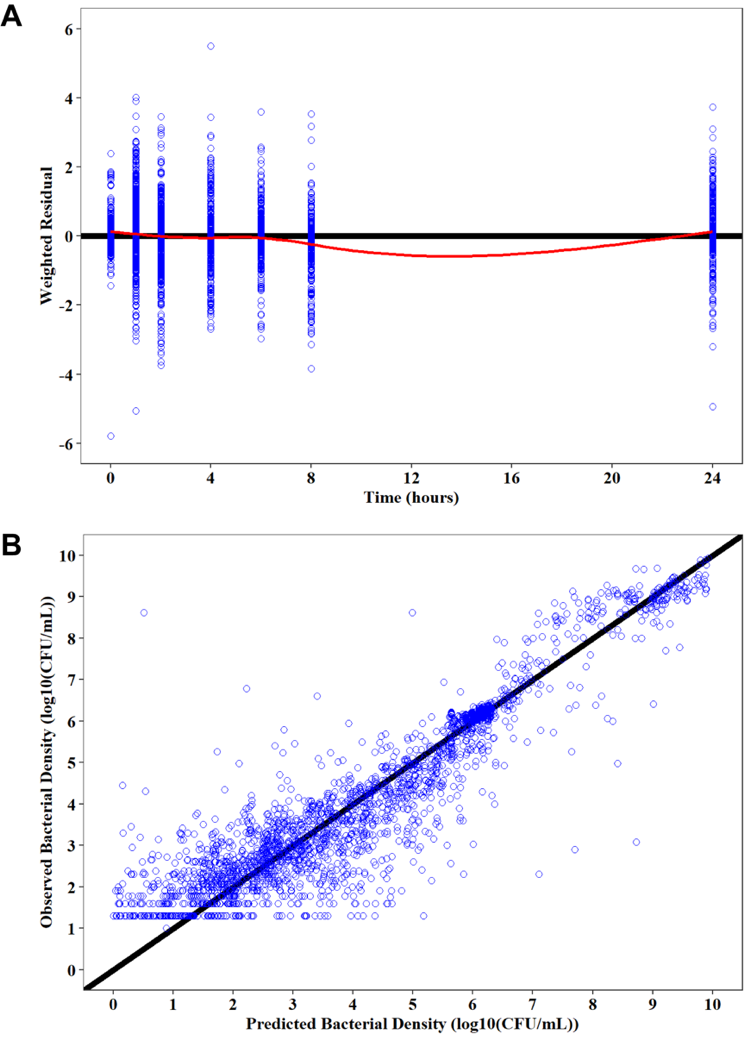


**Supplemental Figure S9.** Diagnostic plots for evaluating model performance of the Mechanism-Based Model for all 22 isolates within the secondary cohort. (A) weighted residuals versus time and (B) observed versus predicted bacterial density. Observed bacterial counts are bounded by the limit of detection (LOD) during plated counting, while the predicted bacterial counts can extend below the LOD

**References**

1. Chow JW, Yu VL. 1999. Combination antibiotic therapy versus monotherapy for gram-negative bacteraemia: a commentary. Int J Antimicrob Agents 11:7-12.

2. Charlson ME, Pompei P, Ales KL, MacKenzie CR. 1987. A new method of classifying prognostic comorbidity in longitudinal studies: development and validation. J Chronic Dis 40:373-83.

3. van Duin D, Perez F, Rudin SD, Cober E, Hanrahan J, Ziegler J, Webber R, Fox J, Mason P, Richter SS, Cline M, Hall GS, Kaye KS, Jacobs MR, Kalayjian RC, Salata RA, Segre JA, Conlan S, Evans S, Fowler VG, Jr., Bonomo RA. 2014. Surveillance of carbapenem-resistant Klebsiella pneumoniae: tracking molecular epidemiology and outcomes through a regional network. Antimicrob Agents Chemother 58:4035-41.

4. CLSI. 2021. Performance standards for antimicrobial susceptibility testing, 31st Edition, Wayne, PA, .

5. The European Committee on Antimicrobial Susceptibility Testing. Breakpoint tables for interpretation of MICs and zone diameters, version 11.0, 2021.

6. Bergen PJ, Forrest A, Bulitta JB, Tsuji BT, Sidjabat HE, Paterson DL, Li J, Nation RL. 2011. Clinically relevant plasma concentrations of colistin in combination with imipenem enhance pharmacodynamic activity against multidrug-resistant Pseudomonas aeruginosa at multiple inocula. Antimicrob Agents Chemother 55:5134-42.

7. Jia B, Raphenya AR, Alcock B, Waglechner N, Guo P, Tsang KK, Lago BA, Dave BM, Pereira S, Sharma AN, Doshi S, Courtot M, Lo R, Williams LE, Frye JG, Elsayegh T, Sardar D, Westman EL, Pawlowski AC, Johnson TA, Brinkman FS, Wright GD, McArthur AG. 2017. CARD 2017: expansion and model-centric curation of the comprehensive antibiotic resistance database. Nucleic Acids Res 45:D566-D573.

8. Zankari E, Hasman H, Cosentino S, Vestergaard M, Rasmussen S, Lund O, Aarestrup FM, Larsen MV. 2012. Identification of acquired antimicrobial resistance genes. J Antimicrob Chemother 67:2640-4.

9. Chen L, Zheng D, Liu B, Yang J, Jin Q. 2016. VFDB 2016: hierarchical and refined dataset for big data analysis--10 years on. Nucleic Acids Res 44:D694-7.

10. Carattoli A, Zankari E, Garcia-Fernandez A, Voldby Larsen M, Lund O, Villa L, Moller Aarestrup F, Hasman H. 2014. In silico detection and typing of plasmids using PlasmidFinder and plasmid multilocus sequence typing. Antimicrob Agents Chemother 58:3895-903.

11. Deleo FR, Chen L, Porcella SF, Martens CA, Kobayashi SD, Porter AR, Chavda KD, Jacobs MR, Mathema B, Olsen RJ, Bonomo RA, Musser JM, Kreiswirth BN. 2014. Molecular dissection of the evolution of carbapenem-resistant multilocus sequence type 258 Klebsiella pneumoniae. Proc Natl Acad Sci U S A 111:4988-93.

12. Arndt D, Grant JR, Marcu A, Sajed T, Pon A, Liang Y, Wishart DS. 2016. PHASTER: a better, faster version of the PHAST phage search tool. Nucleic Acids Res 44:W16-21.

13. Delcher AL, Salzberg SL, Phillippy AM. 2003. Using MUMmer to identify similar regions in large sequence sets. Curr Protoc Bioinformatics Chapter 10:Unit 10 3.

14. Croucher NJ, Page AJ, Connor TR, Delaney AJ, Keane JA, Bentley SD, Parkhill J, Harris SR. 2015. Rapid phylogenetic analysis of large samples of recombinant bacterial whole genome sequences using Gubbins. Nucleic Acids Res 43:e15.

15. Lam MMC, Wick RR, Watts SC, Cerdeira LT, Wyres KL, Holt KE. 2021. A genomic surveillance framework and genotyping tool for Klebsiella pneumoniae and its related species complex. Nat Commun 12:4188.

16. Price MN, Dehal PS, Arkin AP. 2009. FastTree: computing large minimum evolution trees with profiles instead of a distance matrix. Mol Biol Evol 26:1641-50.

17. Rambaut A. 2009. FigTree version 1.3.1.

18. Krzywinski M, Schein J, Birol I, Connors J, Gascoyne R, Horsman D, Jones SJ, Marra MA. 2009. Circos: an information aesthetic for comparative genomics. Genome Res 19:1639-45.

19. Sharma R, Patel S, Abboud C, Diep J, Ly NS, Pogue JM, Kaye KS, Li J, Rao GG. 2017. Polymyxin B in combination with meropenem against carbapenemase-producing Klebsiella pneumoniae: pharmacodynamics and morphological changes. Int J Antimicrob Agents 49:224-232.

20. Rees VE, Bulitta JB, Oliver A, Nation RL, Landersdorfer CB. 2019. Evaluation of Tobramycin and Ciprofloxacin as a Synergistic Combination Against Hypermutable Pseudomonas Aeruginosa Strains via Mechanism-Based Modelling. Pharmaceutics 11.

21. Rao GG, Landersdorfer CB. 2021. Antibiotic pharmacokinetic/pharmacodynamic modelling: MIC, pharmacodynamic indices and beyond. Int J Antimicrob Agents 58:106368.

22. Garonzik SM, Li J, Thamlikitkul V, Paterson DL, Shoham S, Jacob J, Silveira FP, Forrest A, Nation RL. 2011. Population pharmacokinetics of colistin methanesulfonate and formed colistin in critically ill patients from a multicenter study provide dosing suggestions for various categories of patients. Antimicrob Agents Chemother 55:3284-94.

23. Nation RL, Garonzik SM, Li J, Thamlikitkul V, Giamarellos-Bourboulis EJ, Paterson DL, Turnidge JD, Forrest A, Silveira FP. 2016. Updated US and European Dose Recommendations for Intravenous Colistin: How Do They Perform? Clin Infect Dis 62:552-558.

24. Nation RL, Garonzik SM, Thamlikitkul V, Giamarellos-Bourboulis EJ, Forrest A, Paterson DL, Li J, Silveira FP. 2017. Dosing guidance for intravenous colistin in critically-ill patients. Clin Infect Dis 64:565-571.

25. GlaxoSmithKline Manufacturing Spa. AVYCAZ [package insert]. U.S. Food and Drug Administration website. https://www.accessdata.fda.gov/drugsatfda_docs/label/2019/206494s005,s006lbl.pdf. Accessed October 26, 2020

26. Li J, Lovern M, Green ML, Chiu J, Zhou D, Comisar C, Xiong Y, Hing J, MacPherson M, Wright JG, Riccobene T, Carrothers TJ, Das S. 2019. Ceftazidime-Avibactam Population Pharmacokinetic Modeling and Pharmacodynamic Target Attainment Across Adult Indications and Patient Subgroups. Clin Transl Sci 12:151-163.

27. Cheah SE, Wang J, Nguyen VT, Turnidge JD, Li J, Nation RL. 2015. New pharmacokinetic/pharmacodynamic studies of systemically administered colistin against Pseudomonas aeruginosa and Acinetobacter baumannii in mouse thigh and lung infection models: smaller response in lung infection. J Antimicrob Chemother 70:3291-7.

28. Ly NS, Bulitta JB, Rao GG, Landersdorfer CB, Holden PN, Forrest A, Bergen PJ, Nation RL, Li J, Tsuji BT. 2015. Colistin and doripenem combinations against Pseudomonas aeruginosa: profiling the time course of synergistic killing and prevention of resistance. J Antimicrob Chemother 70:1434-42.

29. Bulitta JB, Yang JC, Yohonn L, Ly NS, Brown SV, D'Hondt RE, Jusko WJ, Forrest A, Tsuji BT. 2010. Attenuation of colistin bactericidal activity by high inoculum of Pseudomonas aeruginosa characterized by a new mechanism-based population pharmacodynamic model. Antimicrob Agents Chemother 54:2051-62.

30. Rao GG, Ly NS, Bulitta JB, Soon RL, San Roman MD, Holden PN, Landersdorfer CB, Nation RL, Li J, Forrest A, Tsuji BT. 2016. Polymyxin B in combination with doripenem against heteroresistant Acinetobacter baumannii: pharmacodynamics of new dosing strategies. J Antimicrob Chemother 71:3148-3156.

31. Bulitta JB, Ly NS, Yang JC, Forrest A, Jusko WJ, Tsuji BT. 2009. Development and qualification of a pharmacodynamic model for the pronounced inoculum effect of ceftazidime against Pseudomonas aeruginosa. Antimicrob Agents Chemother 53:46-56.

32. Tait JR, Bilal H, Kim TH, Oh A, Peleg AY, Boyce JD, Oliver A, Bergen PJ, Nation RL, Landersdorfer CB. 2021. Pharmacodynamics of ceftazidime plus tobramycin combination dosage regimens against hypermutable Pseudomonas aeruginosa isolates at simulated epithelial lining fluid concentrations in a dynamic in vitro infection model. J Glob Antimicrob Resist 26:55-63.

33. Lin YW, Yu HH, Zhao J, Han ML, Zhu Y, Akter J, Wickremasinghe H, Walpola H, Wirth V, Rao GG, Forrest A, Velkov T, Li J. 2018. Polymyxin B in Combination with Enrofloxacin Exerts Synergistic Killing against Extensively Drug-Resistant Pseudomonas aeruginosa. Antimicrob Agents Chemother 62.

34. Landersdorfer CB, Ly NS, Xu H, Tsuji BT, Bulitta JB. 2013. Quantifying subpopulation synergy for antibiotic combinations via mechanism-based modeling and a sequential dosing design. Antimicrob Agents Chemother 57:2343-51.

35. Maidhof H, Johannsen L, Labischinski H, Giesbrecht P. 1989. Onset of penicillin-induced bacteriolysis in staphylococci is cell cycle dependent. J Bacteriol 171:2252-7.

36. Mould DR, Upton RN. 2013. Basic concepts in population modeling, simulation, and model-based drug development-part 2: introduction to pharmacokinetic modeling methods. CPT Pharmacometrics Syst Pharmacol 2:e38.

37. Rao GG, Li J, Garonzik SM, Nation RL, Forrest A. 2018. Assessment and modelling of antibacterial combination regimens. Clin Microbiol Infect 24:689-696.

38. Nation RL, Li J. 2009. Colistin in the 21st century. Curr Opin Infect Dis 22:535-43.

39. Yadav R, Landersdorfer CB, Nation RL, Boyce JD, Bulitta JB. 2015. Novel approach to optimize synergistic carbapenem-aminoglycoside combinations against carbapenem-resistant Acinetobacter baumannii. Antimicrob Agents Chemother 59:2286-98.

40. Yadav R, Bulitta JB, Nation RL, Landersdorfer CB. 2017. Optimization of Synergistic Combination Regimens against Carbapenem- and Aminoglycoside-Resistant Clinical Pseudomonas aeruginosa Isolates via Mechanism-Based Pharmacokinetic/Pharmacodynamic Modeling. Antimicrob Agents Chemother 61.

41. Bauer RJ, Guzy S, Ng C. 2007. A survey of population analysis methods and software for complex pharmacokinetic and pharmacodynamic models with examples. AAPS J 9:E60-83.

42. Bulitta JB, Bingolbali A, Shin BS, Landersdorfer CB. 2011. Development of a new pre- and post-processing tool (SADAPT-TRAN) for nonlinear mixed-effects modeling in S-ADAPT. AAPS J 13:201-11.

43. Kuhn M. 2020. caret: Classification and Regression Training. R package version 60-86 https://cran.r-project.org/web/packages/caret/caret.pdf.

44. Marvin N. Wright AZ. 2017. ranger: A Fast Implementation of Random Forests for High Dimensional Data in C++ and R. Journal of Statistical Software 77:1-17.

45. Breiman L. 2001. Random Forest. Machine Learning 45:5-32.

46. Diaz-Uriarte R, Alvarez de Andres S. 2006. Gene selection and classification of microarray data using random forest. BMC Bioinformatics 7:3.

47. Uddin S, Khan A, Hossain ME, Moni MA. 2019. Comparing different supervised machine learning algorithms for disease prediction. BMC Med Inform Decis Mak 19:281.

48. Kegerreis B, Catalina MD, Bachali P, Geraci NS, Labonte AC, Zeng C, Stearrett N, Crandall KA, Lipsky PE, Grammer AC. 2019. Machine learning approaches to predict lupus disease activity from gene expression data. Sci Rep 9:9617.

49. Robinson GA, Peng J, Donnes P, Coelewij L, Naja M, Radziszewska A, Wincup C, Peckham H, Isenberg DA, Ioannou Y, Pineda-Torra I, Ciurtin C, Jury EC. 2020. Disease-associated and patient-specific immune cell signatures in juvenile-onset systemic lupus erythematosus: patient stratification using a machine-learning approach. Lancet Rheumatol 2:e485-e496.

50. Long NP, Park S, Anh NH, Min JE, Yoon SJ, Kim HM, Nghi TD, Lim DK, Park JH, Lim J, Kwon SW. 2019. Efficacy of Integrating a Novel 16-Gene Biomarker Panel and Intelligence Classifiers for Differential Diagnosis of Rheumatoid Arthritis and Osteoarthritis. J Clin Med 8.

51. Huang X, Pan W, Grindle S, Han X, Chen Y, Park SJ, Miller LW, Hall J. 2005. A comparative study of discriminating human heart failure etiology using gene expression profiles. BMC Bioinformatics 6:205.

52. Wu B, Abbott T, Fishman D, McMurray W, Mor G, Stone K, Ward D, Williams K, Zhao H. 2003. Comparison of statistical methods for classification of ovarian cancer using mass spectrometry data. Bioinformatics 19:1636-43.

53. Arbabshirani MR, Plis S, Sui J, Calhoun VD. 2017. Single subject prediction of brain disorders in neuroimaging: Promises and pitfalls. Neuroimage 145:137-165.

54. Vabalas A, Gowen E, Poliakoff E, Casson AJ. 2019. Machine learning algorithm validation with a limited sample size. PLoS One 14:e0224365.

55. Varma S, Simon R. 2006. Bias in error estimation when using cross-validation for model selection. BMC Bioinformatics 7:91.
